# The impact of stress and anesthesia on animal models of infectious disease

**DOI:** 10.3389/fvets.2023.1086003

**Published:** 2023-02-02

**Authors:** Rachel Layton, Daniel Layton, David Beggs, Andrew Fisher, Peter Mansell, Kelly J. Stanger

**Affiliations:** ^1^Australian Centre for Disease Preparedness, CSIRO, Geelong, VIC, Australia; ^2^Faculty of Veterinary and Agricultural Sciences, Melbourne Veterinary School, University of Melbourne, Melbourne, VIC, Australia

**Keywords:** infectious disease research, laboratory animal welfare, impacts of anesthesia, impacts of stress, animal monitoring, animal models of disease, animal immunity, surgical stress

## Abstract

Stress and general anesthesia have an impact on the functional response of the organism due to the detrimental effects on cardiovascular, immunological, and metabolic function, which could limit the organism's response to an infectious event. Animal studies have formed an essential step in understanding and mitigating infectious diseases, as the complexities of physiology and immunity cannot yet be replicated *in vivo*. Using animals in research continues to come under increasing societal scrutiny, and it is therefore crucial that the welfare of animals used in disease research is optimized to meet both societal expectations and improve scientific outcomes. Everyday management and procedures in animal studies are known to cause stress, which can not only cause poorer welfare outcomes, but also introduces variables in disease studies. Whilst general anesthesia is necessary at times to reduce stress and enhance animal welfare in disease research, evidence of physiological and immunological disruption caused by general anesthesia is increasing. To better understand and quantify the effects of stress and anesthesia on disease study and welfare outcomes, utilizing the most appropriate animal monitoring strategies is imperative. This article aims to analyze recent scientific evidence about the impact of stress and anesthesia as uncontrolled variables, as well as reviewing monitoring strategies and technologies in animal models during infectious diseases.

## Introduction

The complex interplay of the immune system and physiology of infection can't be replicated *in vitro* and is very limited in *ex vivo* studies (1), meaning animal models are still essential to the study of infectious disease (2). Animal models are used to study infectious diseases in both human and veterinary medicine, but the results of these studies are vulnerable to a series of variables such as handling, cage environment, and technical procedures, which can generate varying degrees of stress (3). The physiological and immunological consequences of stress, in addition to other factors such as the induction of general anesthesia, have the potential to alter scientific outcomes resulting in less applicable science (4). Additional consequences of these uncontrolled variables in infectious disease research are poorer animal welfare outcomes (5). There is increasing societal scrutiny and expectations on how animal research is conducted by the general public, with increasing expectations that research involving animals is both well-justified and conducted in a manner that not only minimizes animal suffering but results in an overall positive welfare experience (6). Continually improving the applicability of science from the laboratory to real-world application is therefore crucial, in addition to enhancing animal welfare by adapting and further developing best-practice methods of laboratory animal care and management (5). Both objectives can be achieved *via* the identification and reduction of study variables.

Of the many variables that can impact upon studies of infectious disease, the effects of stress on immunity and disease susceptibility are well-documented (7, 8). Stress is a complex and multi-faceted process (chronic vs. acute, beneficial vs. adverse effects) and consequently, there are inherent difficulties in identifying what causes stress in different species under diverse study conditions (9). Stress experienced by animals in disease research can be caused by the disease itself and accompanying inflammatory responses (10, 11), as well as regular animal handling and repeated procedures and interventions (3–5, 12). The factors that cause stress also promote the organism's response as a homeostasis-related compensatory mechanism or returning to homeostasis, by modifying the physiological parameters and generating compensatory metabolic, hormonal or neurological responses that can alter study results (13–16). The impacts of stress can be detrimental to both animal welfare and scientific outcomes in animal models (17), but stress is certainly not the only significant cause of study variables in infectious disease research.

The administration of sedatives and anesthetics is a common requirement in animal studies for sample collection (18). Yet despite its accepted and regular use in animal studies of infectious disease, general anesthesia has multi-modal effects on immune system functioning (19). Although general anesthetics are known to interfere with the immune system causing immunosuppression, repeated and regular anesthetic events commonly occur throughout animal studies. Whilst the use of anesthesia plays a crucial role in the effective management of animal welfare and meeting scientific objectives, potential immunomodulatory effects of anesthetic induction should not be ignored. In addition, the induction of anesthesia often introduces its own negative impacts on animal welfare such as cognitive dysfunction (20). This dysfunction can present as a decrease in learning, memory capacity or inability to concentrate, only if the appearance of central inflammation and neuronal apoptosis is induced, where synaptic loss could promote neuroinflammation (21).

Accurately quantifying the impacts of stress and anesthesia as variables in animal models of infectious disease relies on the methods of assessment being used. In addition to the more traditional clinical and subjective assessment methods, recent developments in non-invasive monitoring technology are beginning to be adapted and utilized for the collection of physiological data in animal studies of disease. This includes the measure of heart rate and heart rate variability in rodent stroke models (22), the use of collar monitors for the identification of subclinical mastitis in dairy cattle (23), and the detection of respiratory disease in pigs using infra-red and conventional imaging (24). This multi-faceted monitoring approach leads to an improved understanding of disease, enhanced animal welfare *via* monitoring and humane endpoint refinement, and the potential to more effectively identify and mitigate the detrimental effects of stress and anesthesia on infectious disease study outcomes (25).

This review aims to describe how stress and anesthesia act as uncontrolled variables that impact upon scientific and animal welfare outcomes in animal studies of infectious disease. It discusses how the effects of stress and anesthesia can be understood and addressed during the planning and conduct of *in vivo* infectious disease studies, and presents novel recommendations for future research to better understand and mitigate the physiological and immunological impacts of stress, pain, and anesthesia. Current and emerging monitoring strategies and technologies to assess animal health and disease most effectively are described. In addition, this review presents recommendations for the future refinement and enhanced uptake of optimized monitoring strategies in experimental animal models of infectious disease.

## Methodology

Literature was searched *via* PubMed, Google Scholar, and Scopus *via* keyword searches for all topics reviewed. For the analysis of animal monitoring methods between 2012/2013 to 2020/2021 a search of title, abstract, and key words on web of science was conducted using the following search categories: Veterinary Sciences, Infectious Diseases, Agriculture multidisciplinary, and Zoology. Animal monitoring methods were categorized and search terms used as described in Table 1.

**Table 1:** Categories of animal monitoring and search terms used per category.

	**Machine learning and algorithms**	**Subjective assessment and operator scoring**	**Clinical parameter assessment**	**Sensors and wearable devices**	**Video monitoring**
**Search terms used within category**	- Machine learning - Machine learning animal disease - Algorithm animal disease	- Subjective assessment animal disease - Clinical scoring animal disease - Grimace score animal disease - Operator assessment animal disease	- Heart rate animal disease - Rectal temperature animal disease - Blood pressure animal disease - Respiration animal disease	- Sensors animal disease - Wearable animal disease	- Video monitoring animal disease - Infrared monitoring animal disease- Motion detection monitoring animal disease

### The impact of stress on infectious disease study outcomes

In the context of laboratory animals stress can be defined as a negative emotional experience accompanied by predictable biochemical, physiological, cognitive, and behavioral changes that are directed either toward altering the stressful event or accommodating to its effects (26). This definition is in line with the founding principles of humane animal research developed by Russel and Burch (27), which defines distress in laboratory animals as a central nervous state of a certain rank on a scale, in the direction of the mass autonomic response which if protracted, would lead to the physiologic stress syndrome (27). Animals maintained in laboratory conditions are often far removed from their evolved or natural environment, and this can predispose these animals to experiencing greater levels of stress (4). In addition, keeping animals in controlled environments away from stress factors may predispose animals to experience a greater degree of stress, resulting in neurobiological, hormonal, and metabolic compensatory responses that result in the development of chronic stress (16, 28).

The effects of chronic stress on immunity and disease susceptibility in humans and animals is well-established in the literature, as demonstrated in a study by Cohen et al. (29). The authors experimentally exposed healthy human volunteers to rhinoviruses with varied histories of experience with chronic stressors. Their results showed those individuals with recent long-term exposure to a threatening stressful experience demonstrated glucocorticoid receptor resistance and were at higher risk of succumbing to a viral infection. In addition, glucocorticoid receptor resistance predicted the production of higher levels of pro-inflammatory cytokines and disease among infected subjects. This is a clear demonstration of not only the effects of chronic stress on increasing the risk of disease susceptibility, but also the mechanisms that lead to reduced disease resistance. Zhou et al. (30) have also demonstrated compromised immunity due to chronic stress in animal cancer models. The authors applied chronic mild stress to mice with cancerous tumors undergoing immunotherapy and found that tumor regression occurred in mice undergoing immunotherapy, but this regression was attenuated in mice undergoing mild chronic stress (30). These results have implications for infectious disease research, where compromised immunity can result in altered disease outcomes.

Such altered disease outcomes were demonstrated by Gervasi et al. (31). The authors experimentally altered levels of the stress hormone corticosteroid *via* a hormone implant in two groups of zebra finches (10 finches implanted with low corticosterone devices and 10 finches implanted with high corticosterone devices), with a third control group of 10 finches not receiving any corticosteroid implants. Blood was collected from all finches prior to exposure to West Nile virus, with the average corticosterone level of control birds being ~15 ng/ml, low dose corticosterone group birds ~50 ng/ml, and high dose corticosterone group birds ~100 ng/ml (31). They found that although all birds became infected, only birds with elevated corticosteroid had viral loads at or above the infectious threshold. Further, no mortality was observed in control birds, whilst mortality rates of 40 and 70% were observed in low corticosterone and high corticosterone implanted finches, respectively. This suggests that immunosuppression caused by elevated glucocorticoid stress hormones leads to a higher susceptibility to disease. In a similar study in mice, Zhou et al. (32) inoculated cancer cells into mammary fat pads of control, stress, and stress + chewing groups where mice were provided wooden blocks to chew on whilst undergoing psychosocial stress. They found that psychosocial stress enhanced tumor growth, but chewing behavior markedly inhibited this growth by ameliorating the effects of stress, and in turn modulating stress hormones and their receptors (32). This highlights the importance of identifying and reducing chronic stressors in animal studies in order to prevent the development of physiological compensatory mechanisms that lead to chronic stress responses, such as impaired immune and altered metabolic, neurobiological, and cardiovascular functions (16, 28). As laboratory animal stress can result from many common animal husbandry, environmental, and procedural factors, there is considerable potential for everyday stressors to impact upon scientific outcomes in infectious disease studies (33).

**Figure 1:**
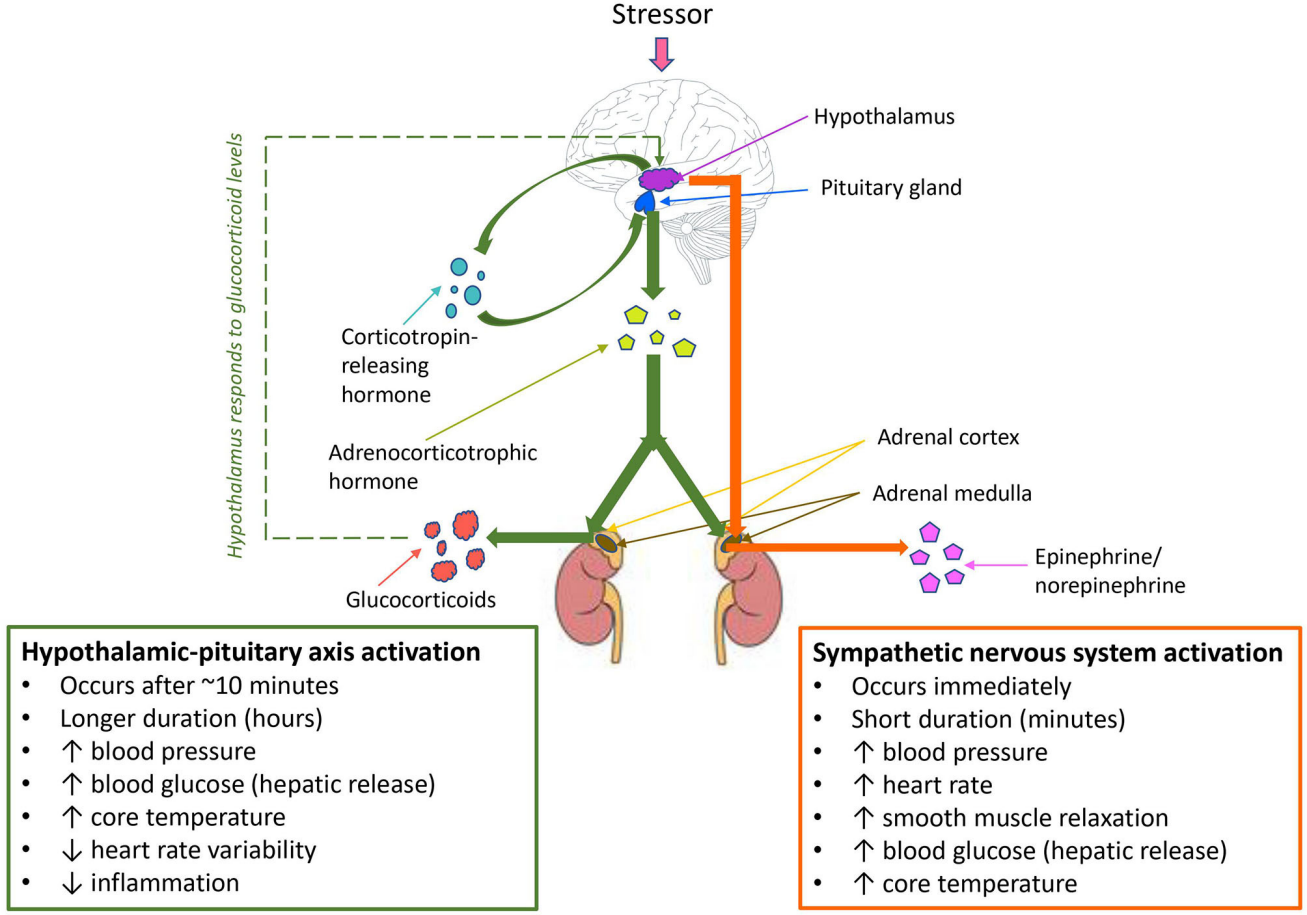
Hypothalamic-pituitary axis (HPA) activation, sympathetic nervous system activation, and compensatory mechanisms in response to stress. Sympathetic nervous system activation occurs as the primary response to stress, followed by HPA axis activation where the stressor is prolonged or chronic. A negative feedback loop leads to compensatory mechanisms *via* the HPA axis in response to threats to homeostasis.

Manual handling and blood collection are two such stressors that are known to cause stress-induced immunomodulation in laboratory animals. Balcombe et al. analyzed data from 80 published *in vivo* studies and determined changes in physiologic parameters correlated with stress (including elevated serum corticosterone, glucose, blood pressure, and heart rate) were associated with both manual handling and blood collection (34). Similar stress responses in mice resulting from noise pollution were also observed in a study by Jafari et al. (35). The authors compared glucocorticoid responses in 32 mice exposed to daily traffic noise (16 mice exposed daily during the light cycle, 16 mice exposed daily during the dark cycle) and equal numbers of control mice not exposed to noise. They found that mice exposed to traffic noise had significantly higher glucocorticoid levels than mice not exposed to traffic noise during both light and dark cycles, regardless of sex (35). Noise sources and levels in animal laboratories are numerous and varied, and can commonly include music, human voices, incidental noise from animal husbandry, and vocalization from other animals in the room (including distress vocalization). The demonstration of elevated glucocorticoid responses in mice exposed to traffic noise indicates the ease in which noise can act as a chronic stressor that has the potential to influence study outcomes and compromise animal welfare. This highlights the importance of identifying and mitigating chronic animal stressors in the laboratory, and indicates that the early recognition of stress factors could help to prevent, control, and diminish the effect of these elements as study variables. A study by Marcon et al. (36) investigated the effects of environmental enrichment of zebrafish responses to chronic stress. The authors submitted two groups of zebra fish, housed with or without environmental enrichment, to unpredictable chronic stress. They found that environmental enrichment attenuated the effects of chronic stress, with zebrafish provided with environmental enrichment displaying significantly less anxiety-like behaviors and reduced cortisol and reactive oxygen species compared to controls with no environmental enrichment (36). In all of these studies, the mechanism of activation of stress responses was found to be directly *via* the hypothalamic-pituitary-adrenal (HPA) axis in the form of enhanced production of glucocorticoids, or *via* neural network changes over time in response to enhanced activation of sympathetic nervous activity and chronic exposure to glucocorticoids, rendering glucocorticoid responses more sensitive to stress as described in Figure 1 (16, 28, 37–39).

**Figure 2:**
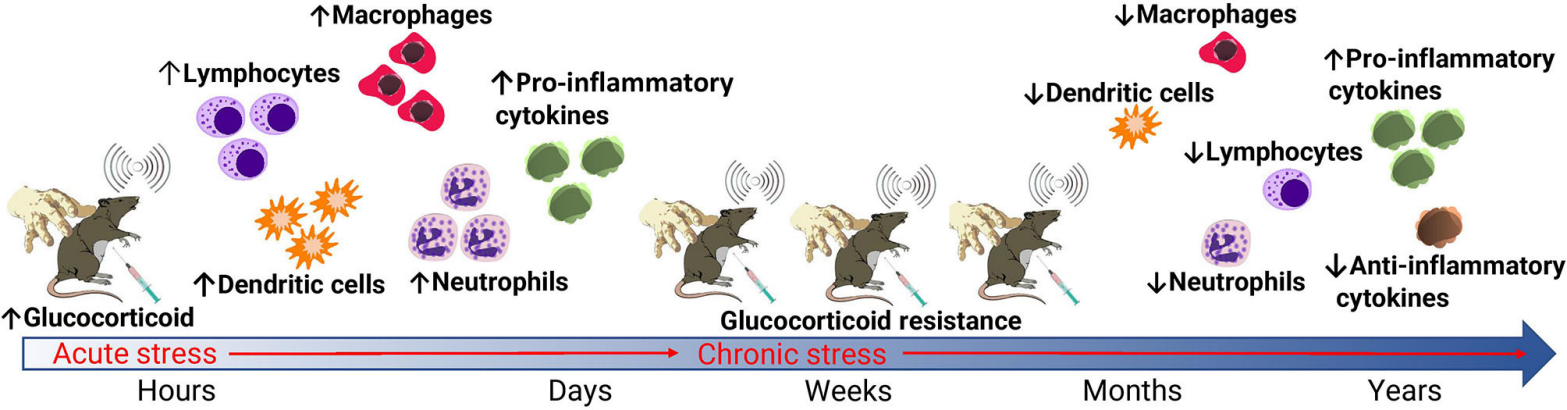
Effects of acute and chronic stress on glucocorticoid and immune responses in laboratory animals. Common routine stressors of laboratory animals include manual handling, blood collection, and noise. When these stressors are acute, enhanced glucocorticoid production *via* stimulation of the sympathetic nervous system and HPA axis result in an enhanced immune response. When stressors are chronic, the development of glucocorticoid resistance leads to immunosuppression. This primarily occurs *via* a decreased and altered leukocyte production in addition to a reduced production of anti-inflammatory cytokines *via* a negative feedback loop.

The HPA activation and stress responses observed in studies of laboratory animal stressors demonstrate poorer welfare outcomes for laboratory animals experiencing chronic stress. A study by Jin et al. (40) takes this a step further, by directly demonstrating the effects of heat stress on immunity and disease susceptibility in mice. The authors infected mice with H5N1 highly pathogenic avian influenza that were previously held in either thermoneutral conditions or placed under chronic heat stress. They found that mice subjected to chronic heat stress exhibited significantly reduced local immune responses in the respiratory tract, in addition to reduced dendritic cell maturation and reduced mRNA levels of IL-6 and interferon (40). Mortality rate and viral load in lungs was also significantly higher in mice that had experienced chronic heat stress, indicating chronic heat stress caused reduced immunity and increased viral susceptibility. When viewed as a whole, the literature demonstrates substantial and varying impacts of stress on HPA axis activity and immune responses in laboratory animals. When interpreting the effects of stress in the context of animal studies of infectious disease, it is important to consider whether the stressor is likely to be defined as acute or chronic as described in Figure 2 (29, 30, 34, 35, 40, 41). In infectious disease research, both immunosuppressive (commonly resulting from chronic stress) and temporary immunoenhancing /inflammatory (commonly resulting from acute stress) effects are equally important to identify, but understanding the effect is critical for both mitigation of the stressor and interpretation of potential impacts on study results (42). Whilst decreasing laboratory animal stress is crucial for reducing study variables, stress alone is not the only variable that influences outcomes in studies of infectious disease. The administration of preanesthetic drugs and those used for the maintenance of general anesthesia generate a physiological adaptation response that consists of metabolic, neuroendocrine, hemodynamic, immunological, and behavioral changes through the neurosecretion of chemical mediators, which also have the potential to influence the results of infectious disease research (16).

### Physiological impacts of general anesthesia in infectious disease studies

**Figure 3:**
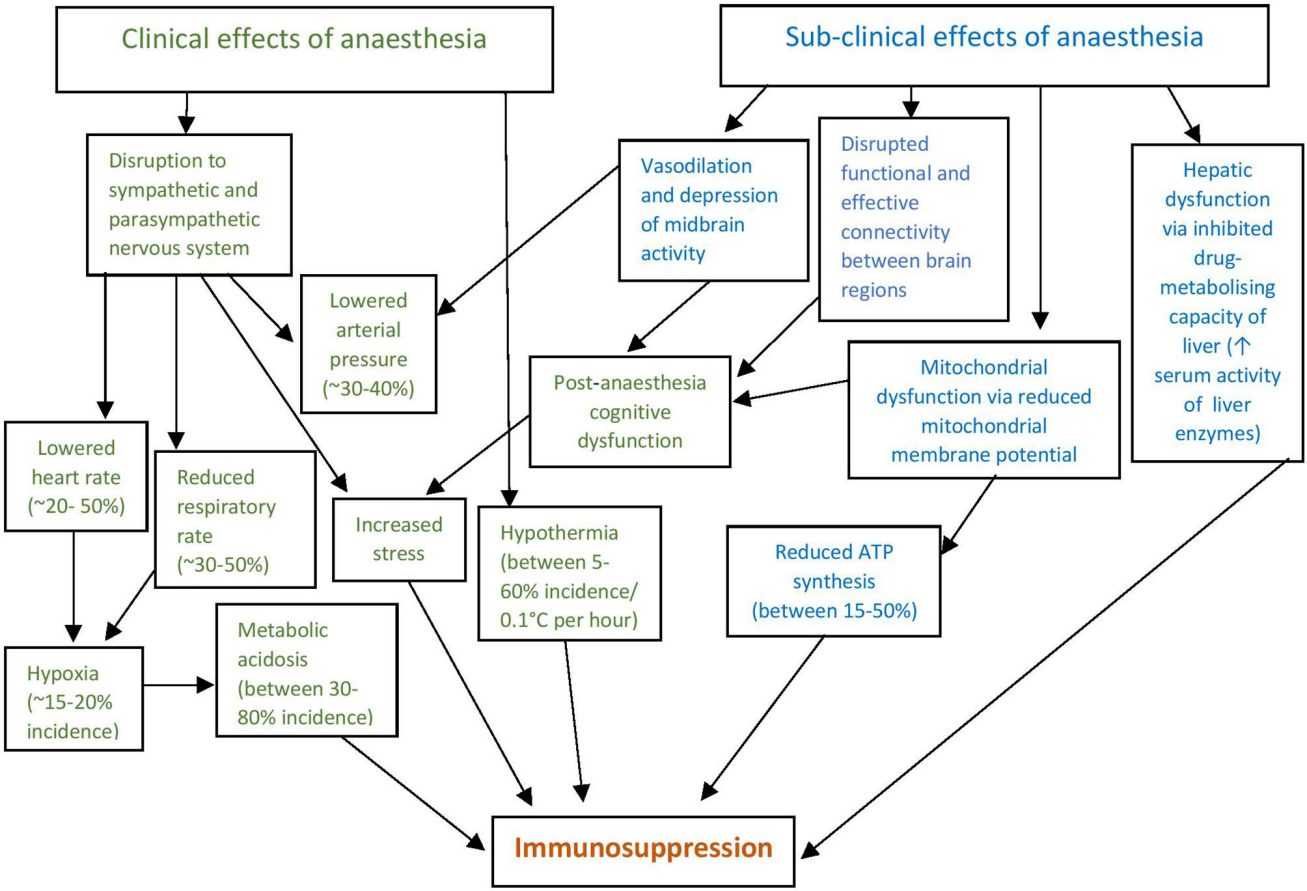
Clinical and subclinical effects of anesthesia lead directly and indirectly to immunosuppression. Clinical effects are those that can be detected by monitoring and assessment, whilst sub-clinical effects are not readily detected. In both instances anesthetic effects either directly result in immunosuppression or result in further physiological effects that in turn result in immunomodulation, most commonly immunosuppression *via* a reduced inflammatory response. The incidence and significance of effects vary depending on the anesthetic agents used, time spent under anesthesia, species being anesthetized and degree of supportive care provided.

Using anesthesia in animal studies of disease can be crucial for the management of animal welfare, operator safety, and the achievement of scientific objectives. This is particularly true for performing invasive procedures, or when conscious restraint or sample collections cause unacceptable stress (43). Whilst the benefits of anesthesia are significant, the use of sedatives, analgesics and anesthetics must be balanced with their own potential risks to animal welfare and altered study outcomes from anesthesia effects (4). Different authors mention that anesthetics can have a depressant effect on the immune, cardiovascular, and metabolic response in healthy animals (44–46). These effects are in addition to behavioral and cognitive deficits, neuroinflammation, and mitochondrial dysfunction (47–52). Figure 3 describes the commonly observed effects of anesthesia (44–46, 48–55).

General anesthesia causes a multitude of physiological effects, which are apparent even in healthy animals. Reductions in arterial pressure of ~30% in healthy dogs have been reported after induction with propofol (5 mg/Kg over 30 s followed by a continuous infusion of 25 mg/Kg/h) (56), as well as medetomidine (0.01 mg/Kg), butorphanol (0.2 mg/Kg) and acepromazine (0.02 mg/Kg) (57). Mrazova et al. (57) further demonstrated an increased respiratory rate and decreased heart rate after fentanyl administration (0.01 mg/Kg), and decreased heart rate and respiratory rate after medetomidine administration in healthy dogs. In sick animals, the effects and potentially detrimental consequences of anesthesia can be exacerbated. A cohort study conducted by Brodbelt et al. (58) surveyed 117 veterinary practices in the United Kingdom, analyzing data obtained from 98,036 dogs, 79,178 cats and 8,209 rabbits that had been anesthetized and sedated using various anesthetic and sedation regimes. The authors found that in healthy animals with no pre-existing disease, the risk of death from anesthesia or sedation was 0.05% for dogs, 0.11% for cats, 0.73% for rabbits, and 3.8% for guinea pigs (58). However, these risks increased dramatically for sick animals-−1.33% (two–three-fold increase) for dogs, 1.40% (12–13-fold increase) for cats, and 7.37% (10-fold increase) for rabbits. This dramatic increase in mortality risk in sick compared to healthy animals illustrates the significantly enhanced impacts of anesthesia on compromised animals. Given that disease research models typically result in illness, the impact of anesthesia on animal welfare and resultant study outcomes in disease research is of concern. More specifically, the effects of a standard ketamine/xylazine mouse anesthetic regime were investigated by Schuetze et al. (59). By anesthetizing both young (2.14 ± 0.23 months) and aged (26.31 ± 2.15 months) mice with a standard dose, the authors found that 0 of the 26 young mice died under anesthesia, compared to 4 out of 26 aged mice (15.4% mortality) (59). In addition to the physiological variables that could be introduced to surviving mice in a disease study, the loss of such a large number of mice in a study can reduce statistical power, risking the ability to achieve study objectives (60). Studies into the mortality of commonly used research species that are not commonly anesthetized in veterinary practice, such as pigs, are needed to more accurately quantify the mortality rate of anesthesia in these species. This will allow for further understanding of anesthetic risk levels, which is important for study design and improved research animal management practices (61).

An important consequence of general anesthesia is a reduced ability to thermoregulate and maintain core body temperature within a thermoneutral zone. Contributing factors to this reduced thermoregulatory ability under general anesthesia include vasodilation leading to greater heat loss to the surrounding environment, changes to central brain structure activity, cooling effects of disinfectant application, and heat loss resulting from surgical penetration of the body cavity (62). These factors can be mitigated by the use of management techniques peri- and post-anesthesia such as effective warming and supportive care. However, where this is not optimized, the physiological and physical effects of anesthesia can be exacerbated (63). The ability to provide optimal supportive care to reduce the impacts of anesthesia in research animals is highly variable, and dependent on many factors. The core temperature of smaller animals (such as rodents) is relatively easy to maintain through portable heat mats and lamps and is largely deemed necessary and considered standard practice (64). Even short-term interruptions to thermoregulation from anesthetic induction can easily lead to serious complications or death in smaller species, due in part to the large surface-area-to-volume ratio of small mammals (65). For larger research animals (such as pigs and cattle) the management of anesthesia-associated issues is often more difficult, due to the increased complexity of providing effective warming and other means of supportive care to larger animals in the infectious disease research setting. Rodriguez-Diaz et al. (66) analyzed the incidence of perioperative inadvertent hypothermia in dogs and cats. The authors demonstrated that despite the standard use of warming equipment and supportive care protocols in veterinary practice, a high incidence of perioperative anesthesia-associated hypothermia was identified (66). Given that warming and supportive care for larger animals undergoing anesthesia in infectious disease studies is often less optimized compared to the clinical veterinary setting, it is reasonable to expect the incidence and severity of hypothermia to be even more pronounced than that identified by Rodriguez-Diaz et al. (66). Hypothermia has significant and wide-ranging effects on the immune response, with lowered core temperature driving anti-inflammatory/resolution-type effector functions (67). In animal infectious disease studies, using anesthesia and the associated varying degrees of hypothermia that can result (often at repeated timepoints) is therefore likely to impact upon the immune response to the diseases being studied, but further direct research is required to quantify these potential effects. Adverse neurological effects have been described during an induced hypothermic circulatory arrest for cardiac surgery (68), but this degree of hypothermia (reduction in core temp to 18 degrees) is not seen as a consequence of standard anesthesia (69).

Besides immunomodulation, anesthesia-associated hypothermia results in an increased risk of coagulopathies, most likely to occur *via* two mechanisms- reduced platelet function, and the functional impairment of several enzymes of the coagulation cascade and subsequent reduction in clot formation (70). This has significance for infectious disease studies in general, but even more specifically for the study of diseases that cause coagulopathies. Petrilli et al. (71) demonstrated a mechanism by which coagulopathic infectious disease morbidity outcomes are influenced by disruptions to the coagulation cascade. The authors retrospectively studied patients with COVID-19 and identified that elevated d-dimer levels were strongly associated with critical illness (71). D-dimer is a product of fibrin degradation and is only present in plasma as a result of activation of the coagulation cascade, as occurs after degradation of blood clots (72). The findings of Petrilli et al. (71) therefore demonstrate that increased coagulopathy and clot formation leads to increased morbidity in COVID-19 patients. Building on this observation, Wang et al. (73) studied retrospective cases of COVID-19 patients and found that elevated d-dimer is a significant component of disseminated intravascular coagulation, which develops due to abnormalities with the coagulation cascade and is a leading cause of in-hospital deaths in COVID-19 patients. These findings suggest that anesthesia-associated hypothermia could result in altered study outcomes in SARS-CoV-2 animal studies, by pre-disposing and increasing the susceptibility of research animals to coagulopathies and clot formation which in turn increases mortality risk (74). Additionally, hypothermia induces multiple cardiopulmonary effects including reductions in heart rate, respiratory rate, and systolic blood pressure (75). As COVID-19 can elicit severe acute respiratory syndrome and cardiac and lung injury, changes to cardiopulmonary function as a result of hypothermia may also increase disease susceptibility or lead to alterations in disease course and presentation (76). These effects may also be true for other infectious diseases that can result in coagulopathy such as Ebola, Dengue, and Chikungunya virus, but further investigation is required to determine this.

Whilst there is a distinct lack of research demonstrating how general anesthetic induction specifically affects infectious disease study outcomes, a recent study by Nash (77) reported on the administration of a low pathogenic strain of influenza to mice anesthetized with ketamine/xylazine and to a control group not administered anesthetic. They found that mice not administered anesthetic displayed very mild or no signs of disease, whilst anesthetized mice succumbed to disease (77). This study directly demonstrates the effects of general anesthesia on disease outcomes and shows the need for more direct studies in varied animal models of infectious disease. The impact of anesthesia on disease outcomes in mice was also demonstrated in an earlier study by Penna et al. (78). Mice were anesthetized with either ketamine or halothane and inoculated with a non-lethal Influenza A virus. They found that mice anesthetized with ketamine had higher viral titres 12 h post-inoculation, and a more rapid lung infiltration of neutrophils and monocytes suggesting differences in the recruitment of immunological effector cells (78). This study shows that different types of anesthesia can result in different immune responses, and therefore cause different disease outcomes. As there are multiple anesthetic combinations used in animal models of disease research, the variables and immunomodulation that can be introduced by different anesthetics are therefore many and varied.

### Impacts of anesthesia-induced immunomodulation on infectious disease outcomes

The wide range of anesthetic combinations utilized in animal models of disease makes identifying the effect of every drug combination, on every species and animal strain, an impossible task. Instead, identifying the known immune-altering consequences of drug classes commonly used in infectious disease research demonstrates the wide-ranging impacts of routinely utilized anesthetics.

#### Alpha-2 adrenergic agonists

Commonly used alpha-2 adrenergic agonists include medetomidine, dexmedetomidine, and xylazine, acting on alpha-2 receptors in the central nervous system and peripheral tissues (79). The physiological impacts of these drugs, particularly on the cardiovascular and pulmonary systems, are well-described in laboratory and small animal medicine and most notably include hypotension/hypertension, bradycardia, and decreased cardiac output (80).

Literature on the immune effects of alpha-2 agonists in this field is less abundant yet studies from human patients demonstrate immunomodulation caused by alpha-2 adrenergic agonists (81–83). Wang et al. (84) analyzed 4,842 human surgical patients, approximately half of which were administered dexmedetomidine for anesthesia. They found that patients administered dexmedetomidine had significantly decreased interleukin (IL)-6 and tumor necrosis factor-α (TNFα) in the blood, and increased IL-10 (84). Compared to the control group the authors also found a significant increase in natural killer cells, B cells, CD4^+^ T cells and a significant decrease in CD8 T cells. Additionally, they observed an increase in the ratios of CD4:CD8 T cells. Overall, the administration of dexmedetomidine in the peri-operative period reduced hyper-inflammatory effects of surgery on the immune system, resulting in improved immune functioning. Interestingly, chickens administered clonidine, another alpha adrenergic agonist, at various doses demonstrated that higher clonidine doses resulted in increased circulating B cells and IgG levels (85). As IgG is critical to host protection during infection and virus neutralization (86), the increased levels caused by clonidine may also have an immunoenhancing effect. However, further studies are required to determine the binding mechanisms of the circulating IgG observed to determine this. Studies in sepsis (87) and myocardial injury (88) demonstrate anti-inflammatory effects of dexmedetomidine primarily as a result of reduced cytokine activity. Anti-inflammatory effects of dexmedetomidine in human infectious disease was also demonstrated by Hamilton et al. (89). The authors conducted a retrospective analysis of 214 adult human patients with severe COVID-19 requiring invasive mechanical ventilation and sedation. They found that risk of mortality was 58.2% lower in patients that were administered dexmedetomidine for sedation within 3.4 days of intubation compared to patients that were not (89). In addition to the reduction in pro-inflammatory cytokine production, dexmedetomidine has also been shown to reduce inflammation by suppressing catecholamine release (90, 91) and reducing immune cell activity and recruitment at sites undergoing inflammatory signaling (92, 93). Further studies in animal models are required to ascertain the anti-inflammatory effects of alpha-2 adrenergic agonists on various infectious disease models. Romifidine is an alpha-2 agonist used primarily in horses, and of which physiological effects have been studied and documented in the literature (94). There is an absence of studies on the effects of romifidine on the immune system, therefore the potential effects on infectious disease study outcomes are currently not known.

Alpha-2 agonists are also known to cause neuroendocrine changes, including blocking insulin release from beta cells and elevating blood glucose levels (95). These effects were demonstrated in a study by Connell et al. (96), who monitored blood glucose levels of diabetic and non-diabetic rats anesthetized with xylazine, medetomidine or pentobarbital. The authors found that both medetomidine and xylazine, but not pentobarbital, elicited marked hyperglycemia in non-diabetic rats. A study by Zhu et al. (97) demonstrates how hyperglycemia may impact upon infectious disease study outcomes. The authors conducted a retrospective, multi-centered study of 7,337 human cases of COVID-19, among which 752 had type 2 diabetes (97). They found that well-controlled blood glucose was associated with markedly lower mortality compared to individuals with poorly controlled blood glucose and hyperglycemia. For studies of infectious disease, this suggests that hyperglycemia induced by the use of alpha-2 agonists could alter disease course and severity and impact upon study outcomes. Alpha-2 agonist effects on beta cells also include the suppression of growth hormone and testosterone (98) and changes to serum prolactin, which acts as both a hormone and a cytokine and has been demonstrated to play an important role in autoimmunity (99). The effects of prolactin on infectious disease study outcomes are not known, and further studies are needed to determine both the effects of prolactin on infectious disease susceptibility and the neuroendocrine impacts of alpha-2 agonists in infectious disease studies.

Overall, the literature is increasingly demonstrating that alpha-2 adrenergic agonists have an overall anti-inflammatory effect on immune responses in relation to infectious disease outcomes. It is important to note that the bulk of research published on the immune altering effects of this drug class is based on single use administration. The immunomodulatory effects of repeated or chronic use, as is common in infectious disease animal studies, is not known and warrants further research.

#### NMDA receptor antagonists

N-methyl-D-aspartate (NMDA) receptor antagonists act by blocking NMDA receptors in the brain, which interact with the neurotransmitter glutamate (100). Ketamine is a commonly used NMDA receptor antagonist that is known to have a range of effects on the immune system. Takahashi et al. (101) conducted laparotomies on mice anesthetized with either sevoflurane or ketamine, followed by intraperitoneal administration of *Escherichia coli* to induce septicemia. The authors found that mice administered ketamine had suppressed TNF-α and reduced phagocytosis. Immunosuppressive effects of ketamine were also found in a study by Gao et al. (102), who isolated peripheral blood mononuclear cells from human blood samples and incubated the cells in either the presence or absence of ketamine. They found that ketamine inhibited Th2 cell differentiation, which are a key cell responsible for the regulation of humoral immune responses (102). Braun et al. (103) demonstrated further effects of ketamine *in vitro* by exposing human immune cells to various doses of ketamine. The authors found that ketamine induced apoptosis in lymphocytes *via* the mitochondrial pathway at lower doses, and *via* necrosis at higher concentrations (103). Additionally, a study by Zeng et al. (104) investigated the effects of ketamine both *in vitro* and *in vivo*, and found that ketamine inhibited the maturation of dendritic cells. The mechanism of this dendritic cell inhibition by ketamine was further explored by Laudanski et al. (105), who obtained monocytes from 36 human subjects and stimulated differentiation into immature dendritic cells in the absence or presence of ketamine at (100, 10 or 1 μg/ml for 5 days). The authors found that at 10 μg/ml or higher, ketamine diminished the differentiation of monocytes into immature dendritic cells *in vitro* (105). As a key role of dendritic cells is the presentation of antigen during infection, the results from both Zeng et al. (104) and Laudanski et al. (105) demonstrate ketamine to have immunosuppressive effects.

As ketamine is administered for non-anesthetic purposes such as chronic pain management, immunosuppressive and anti-inflammatory effects of ketamine have also been demonstrated over repeated use (106) including a reduction in pro-inflammatory cytokines IL-6, IL-1, IL-8 and TNF-α (107). Li et al. (108) further explored the effects of ketamine on hippocampal inflammatory cytokines in both acute and chronic administration mouse models. They found that in mice administered ketamine acutely or chronically, IL-1β and IL-6 levels were both elevated in the hippocampus (108). Additionally, levels of TNF-α were elevated in the single dose model, but significantly decreased in mice administered multiple dose or long-term ketamine. This finding of elevated inflammatory cytokines differs from the bulk of literature that demonstrates immunosuppressive effects of ketamine. This may be due to this study measuring hippocampal cytokine levels as opposed to serum levels, as changes in hippocampal cytokines have been shown to not be reflective of serum cytokine profiles (109). Whilst the measurement of hippocampal cytokines is appropriate to the objectives of this study, in the context of infectious disease serum cytokine levels provide a more relevant and accurate measure of cytokine activity due to differences in cytokine perfusion through the blood-brain barrier (110). The downregulation of systemic inflammatory cytokines by ketamine is also supported by the known mechanisms of ketamine-induced immunosuppression, which includes the downregulation of inflammatory cytokine-producing macrophages and associated protein activation factors (111). Ketamine affects a variety of key immune functions, with the literature demonstrating these effects of be overwhelmingly immunosuppressive even as the result of a single dose.

#### Inhalational anesthetics

Inhalational anesthetics provide the benefit of rapid induction and recovery, the ability to swiftly adjust anesthetic depth as required, and their suitability for use in a wide range of companion, laboratory and livestock animal species (112). Rapid recovery from inhalational anesthesia has the potential benefits of reduced physiological impacts (for example, a reduced incidence and severity of hypothermia) (53). However, immunosuppressive effects of commonly used inhalational agents still occur. Isoflurane, and sevoflurane have all been shown to decrease cytokines, neutrophil cell numbers and adhesion, macrophages and phagocytosis, and natural killer cell cytotoxicity (113). All of these immune effects result in isoflurane and sevoflurane being immunosuppressive. Desflurane is another volatile anesthetic that has also been studied for its effects on immune responses, as demonstrated by Kalimeris et al. (114). The authors compared bronchiolar lavage fluid from 27 pigs anesthetized with either desflurane, sevoflurane or propofol (nine pigs per group), or not anesthetized (an additional four pigs). They found that pigs anesthetized with sevoflurane and desflurane had decreased alveolar macrophages and increased lymphocyte counts compared to controls and pigs anesthetized with propofol (114). The results of these authors reaffirm the immunosuppressive effects of inhalational anesthetics on possibly local cellular immunity, which coincided with a study carried out by Woo et al. (115). The authors assessed immune responses in patients undergoing anesthesia with desflurane. They found that patients had higher levels of neutrophils after desflurane anesthesia, providing an immune protective response (115). In contrast to the literature on isoflurane, desflurane and sevoflurane, Arruda et al. (116) collected blood from patients before and after surgery with halothane anesthesia, and found significant increases in proinflammatory cytokines IL-6 and IL-8. The significantly higher degree of inflammation that halothane induces compared to other inhalational anesthetics has resulted in the largely discontinued use of halothane, as it is this increased production of pro-inflammatory cytokines that can lead to halothane-induced liver injury (117). Studies on desflurane indicate a combination of immunosuppressive and immunoenhancing effects, whilst the literature on isoflurane and sevoflurane demonstrates overwhelmingly immunosuppressive effects. The literature demonstrates that halothane has a substantial inflammatory effect on the immune response, leading to hyper-inflammation which can ultimately result in organ damage and a compromised immune response.

#### General anesthesia and viral proliferation

Besides these immunomodulatory effects of specific drug classes, the administration of general anesthetics can directly affect viral proliferation. A key mechanism of how this occurs is *via* changes to the balanced redox state, which shifts toward oxidant conditions during viral infection (118). Alternatively, a shift away from oxidant conditions, due to higher levels of antioxidants as part of the balanced redox stat, have variable effects on viral growth and can result in viral inhibition or facilitation (119). Erbas et al. (120) studied the effect of general anesthetic agents on the oxidant/antioxidant redox balance in human patients after surgery. They found that both sevoflurane and propofol significantly increased antioxidant levels, whilst desflurane significantly increased oxidant levels (120). Therefore, the antioxidant and immunomodulatory effects of desflurane, sevoflurane, and propofol are likely to affect health and disease outcomes and may affect scientific outputs in animal disease studies.

#### Opioids

Opioids act on mu and kappa receptors in differing ways; as agonists (e.g., morphine, fentanyl), agonist-antagonists (e.g., butorphanol), antagonists (e.g., naloxone), or partial agonists (e.g., buprenorphine) (121). The main opioid receptors are expressed by T lymphocytes and macrophages (122), making these immune cells susceptible to modulation by opioids *via* binding to mu receptors present on these cells (123). Morphine is the most used analgesic in humans and is known to have a wide range of immunosuppressive effects (124) but a more commonly used opioid in infectious disease animal studies is buprenorphine. In a study by Filipczak-Bryniarska et al. (125), mice were administered either buprenorphine, morphine or oxycontin and immune responses compared to baseline. The authors found that mice administered buprenorphine had an enhanced humoral immune response *via* B cell activation, compared to a reduced B cell response in mice administered morphine and no B cell response in mice administered oxycontin (125). Allen and Kendall (126) also investigated the immunosuppressive effects of buprenorphine, by inoculating mice with ovalbumin followed by either saline or slow-release buprenorphine. They found that antibody responses between control and treatment groups did not differ, though IL-10 was significantly higher in mice administered slow-release buprenorphine compared to the control group (126). This indicates that whilst buprenorphine did cause some degree of immune suppression *via* an increase in IL-10 (an anti-inflammatory cytokine), the effects on overall immune function was negligible.

Butorphanol is another commonly used opioid in laboratory animal medicine and is known to have dose-dependent anti-inflammatory and immunosuppressive effects (127). One mechanism of the anti-inflammatory action of butorphanol was demonstrated in a study by Luan et al. (128). The authors induced lung tissue injury in mice *via* sepsis resulting from intraperitoneal lipopolysaccharide injection, then administered butorphanol to one group of mice whilst the other group remained untreated. They found that mice administered butorphanol had lower numbers of pro-inflammatory and higher numbers of anti-inflammatory macrophages compared to untreated mice (128). A reduction in pro-inflammatory macrophages result in a reduction in IL-1β, IL-6, and IL-12, whilst an increase in anti-inflammatory macrophages causes an increase in cytokines including IL-10 (129). These cytokines, both pro-inflammatory and anti-inflammatory, play an important role in the immune response to pathogens, particularly for the development in humoral immunity (130). Modulation of cytokines by butorphanol may therefore affect study outcomes in animal models of infectious disease.

The literature shows that opioids, including commonly used veterinary opioids buprenorphine and butorphanol, can cause immunosuppression *via* a reduced production and proliferation of macrophages and T lymphocytes, with a subsequent modulation of cytokines. When combined with the changes to innate immunity *via* macrophage phagocytosis, both buprenorphine and butorphanol can alter the immune response to pathogens in infectious disease studies, potentially altering study outcomes by enhancing disease susceptibility.

Whilst the literature demonstrates that the majority of opioids have overwhelmingly immunosuppressive effects a notable exception is tramadol, an opioid utilized for analgesia. The immunomodulatory effects of tramadol have previously been shown to cause immunoenhancement *via* significantly enhanced NK cell activity and IL-2 production when administered acutely, but with ongoing chronic administration these immune effects disappeared (131). In other studies, the use of tramadol has been shown to preserve, but not stimulate, immune function when compared to other opioids such as morphine (132). This includes *in vitro* studies showing that morphine decreased monocyte phagocytosis but tramadol did not (133); morphine, methadone, and oxycodone inhibited IL-6 production but tramadol did not (134); that NK cell count decreases were less pronounced in gastric patients administered tramadol compared to morphine (135); and that tramadol administration reduced localized oedema and hyperalgesia without affecting immune mechanisms (136). Whilst the majority of literature demonstrates preservation of immune responses by tramadol, particularly with multiple or chronic administration, some studies have also shown immunosuppressive effects. Bastami et al. (137) investigated the *in vitro* effects of various opioids on TNF-α and IL-8 release. They found that tramadol had the greatest inhibitory effects on IL-8 and TNF-α release compared to morphine, ketobemidone and fentanyl (137). In the context of infectious disease research, tramadol demonstrates potential as an analgesic for moderate pain that results in less immunomodulation than other opioids. Further research is required to determine the effects of tramadol on disease presentation and course in animal models of infectious disease.

#### Local anesthetics

Local anesthesia is a useful tool for both the reduction or elimination of pain in minor procedures and as an addition to multi-modal anesthesia in more invasive surgical procedures (138). Local anesthetics work by blocking voltage-gated sodium channels, which suppresses action potentials in excitable tissues and in turn blocks the transmission of pain impulses (139). The effects of amide local anesthetics (including lidocaine and bupivacaine) on immune responses have been demonstrated in studies of human cancer patients. By reducing the pain response post-surgery and reducing the need for opioids, local anesthetics have been shown to reduce the incidence of tumor recurrence (140). In addition, Piegeler et al. (141) demonstrated direct effects of amide local anesthetics on cancer metastases. The authors incubated lung cancer cells with TNF-α in the presence or absence of amide local anesthetics (lidocaine and ropivacaine). They found that both ropivacaine and lidocaine inhibited tumor cell migration and had an anti-inflammatory effect (141). In the context of infectious disease research, an anti-inflammatory response may impact upon study outcomes, with both ropivacaine and lidocaine being shown to reduce TNF-α-induced upregulation of CD11b/CD18 surface expression on polymorphonuclear leukocytes (PMNs) (142). Another study by Kolle et al. (143) compared the effects of lidocaine and bupivacaine *in vitro* on PMNs, and also found a reduction in granulocyte defense mechanisms for both local anesthetics. These findings are likely to be more relevant for some infectious disease models than others; for example, where local anesthesia is applied to sites of viral inoculation, resulting in reduced PMN activity at the sites of viral replication. In most studies, the locally suppressive effects of local anesthetics on PMNs are unlikely to be of concern given the broad and systemic nature of many infectious disease animal models. Overall, local anesthetics are known for their ability to inhibit excessive inflammatory responses, particularly at the regional level, without causing excessive impairment to host immunity (144).

An additional consideration for the use of local anesthetics in animal models of infectious disease is the potential for an overall reduction in study variables introduced by pain or stress. Given that the use of local anesthesia is so effective at reducing both pain and stress responses (145, 146), the potential direct confounding effects are likely less than the indirect confounding effects of pain and stress if local anesthesia is warranted but not used. Further research comparing different anesthetic and animal management regimes (e.g., general vs. local anesthetic) is warranted in animal models of disease to determine the impacts of local anesthesia on infectious disease study outcomes.

#### Non-steroidal anti-inflammatory drugs

The use of non-steroidal anti-inflammatory drugs (NSAIDs) for the management of pain and inflammation in animals has the benefit of reduced immunomodulatory effects compared to corticosteroids, and act by competitively inhibiting the formation of the inflammatory mediator prostaglandin (147). This limiting of prostaglandin formation occurs *via* NSAID inhibition of cyclo-oxygenase enzymes, of which there are three forms; COX-1, a constitutive member of most tissues including gastrointestinal mucosa, platelets, endothelium, kidneys and uterus; COX-2, which is also constitutive but highly restricted under basal conditions but is upregulated significantly during inflammation; and COX-3, which is mainly expressed in the heart and cerebral cortex (148). Meloxicam is a commonly used NSAID in veterinary medicine and research that inhibits COX-2 (149), and has been demonstrated to effect the immune system by enhancing splenocyte IL-2 release and inhibiting the production of TNF-α, IL-10, and IL-4 in mice (150). In contrast, meloxicam has also been shown to increase TNF-α production in guinea pigs, due to the negative feedback control exerted by prostaglandins on TNF-α formation (151). Prostaglandins play a crucial role in immune responses by supporting activation of dendritic cells whilst suppressing their ability to attract naïve, effector and memory T-cells, modulating chemokine production, and inhibiting the attraction of proinflammatory cells while enhancing local accumulation of regulatory T-cells (152). As meloxicam suppresses prostaglandin release (153), this is likely to have at least some degree of immunosuppression during the infectious disease process. However, a study by Kolstad et al. (154) investigated the impacts of meloxicam, administered at the time of immunization, on antibody titres of mice. They found that use of meloxicam to manage immunization side effects did not affect antibody titres (154).

No effects on antibody titres post-immunization were also demonstrated in rabbits administered carprofen, another commonly used NSAID selective for COX-2 inhibition (155). Carprofen has also been shown to reduce TNF-α activity in rats in a subcutaneous pouch inflammatory model (156) and reduce inflammatory cell infiltrates and serum levels of IL-6 in a mouse model of venous thrombosis (157). These results demonstrate that carprofen and meloxicam have similar anti-inflammatory and immune suppressive effects (158), though whether these anti-inflammatory actions result in significant impacts on disease outcomes in wider studies of infectious disease is not known. Robenacoxib, is a NSAID that is highly selective for COX-2, resulting in its high concentration in and targeting of inflamed tissues (159). Robenacoxib at therapeutic levels has been demonstrated to significantly reduce both lameness scores and synovial fluid levels of C-reactive proteins (CRP), a marker of inflammation, in dogs with osteoarthritis, but not significantly affect CRP serum levels (160). This highly selective nature suggests that robenocoxib may introduce less variables as a NSAID for the management of localized pain and inflammation (e.g., post-surgery) followed by infection in some systemic animals models of infectious disease. However, as NSAIDs with higher selectivity for COX-2 have been shown to have higher risk of cardiovascular complications, their use in infectious disease models that induce cardiovascular compromise may increase the risk of these events occurring (161). Whilst non-selective COX-1 and COX-2 inhibiting NSAIDs (such as piroxicam) have a greater risk of gastrointestinal complications such as pain and bleeding due to their inhibition of COX-1 as well as COX-2 enzymes, transdermal delivery has been shown to significantly reduce these side effects (162). These may provide additional options in infectious disease models where cardiovascular impacts of highly selective COX-2 inhibiting NSAIDS may be of concern. Further research investigating and comparing effects of various NSAIDs in infectious disease studies is required to determine this.

The literature demonstrates that whilst the use of NSAIDs does cause immunomodulation, namely immunosuppression, the impacts on study outcomes in infectious disease studies are likely to be varied and at times negligible depending on the study objectives. The timing and use of NSAIDs should therefore be utilized where deemed necessary for the management of animal welfare and the control of potentially more confounding variables such as unresolved pain and excessive inflammation (158).

#### Pain and uncontrolled inflammation in infectious disease studies

Whilst the use of anesthetic and analgesia can cause immunomodulation, literature showing the significant immunosuppressive impacts of both pain and excessive inflammation (often from tissue trauma or surgery) is extensive (163–165). It is therefore crucial in animal research that in an attempt to reduce variables by avoiding the use of anesthesia and analgesia, that potentially more significant variables in the form of uncontrolled pain and inflammation are not introduced (166). Pain in the absence of tissue injury can suppress NK cell activity and mitogen induced cell proliferation (167, 168) and reduced antibody production (169). Surgical trauma, which commonly combines various degrees of tissue trauma and pain, has been well-demonstrated to cause a variety of immunomodulatory issues including the development of systemic inflammatory immune responses, compensatory anti-inflammatory immune responses, and overall immunosuppression resulting in enhanced disease susceptibility (170). It is therefore crucial that pain and excessive inflammation, for example post-surgery, are well-controlled in studies of infectious disease for the protection of animal welfare and reducing study variables. Despite the known immunomodulatory effects, choosing and administering adequate anesthesia and analgesia for the species and procedure should be a paramount consideration. To minimize negative animal welfare impacts and potential effects on study outcomes, care should be taken to select the least invasive procedures and regimes for achieving study objectives, and utilizing multi-model anesthesia and analgesia to reduce reliance on potentially more impactful drug classes such as opioids (171).

Regardless of the mechanism of effect of anesthetics and analgesics on immune functioning, or indeed whether the effect is immunosuppressive or immunoenhancing, their use can impact scientific outcomes in animal models of infectious disease (65). Where anesthesia and analgesia use are deemed essential for achieving scientific objectives, minimizing potentially more impactful variables such as pain and excessive inflammation, protecting animal welfare, and keeping regimes consistent wherever possible is important. Where different anesthetic or analgesic regimes are utilized, an understanding of their potential effects on disease progression and outcomes is crucial for identifying and understanding study impacts. One way of potentially identifying the effects of study variables, such as stress and different anesthesia regimes, is *via* the selection and use of appropriate and sensitive animal monitoring strategies (172, 173).

### Methods of animal disease and welfare assessment

**Figure 4:**
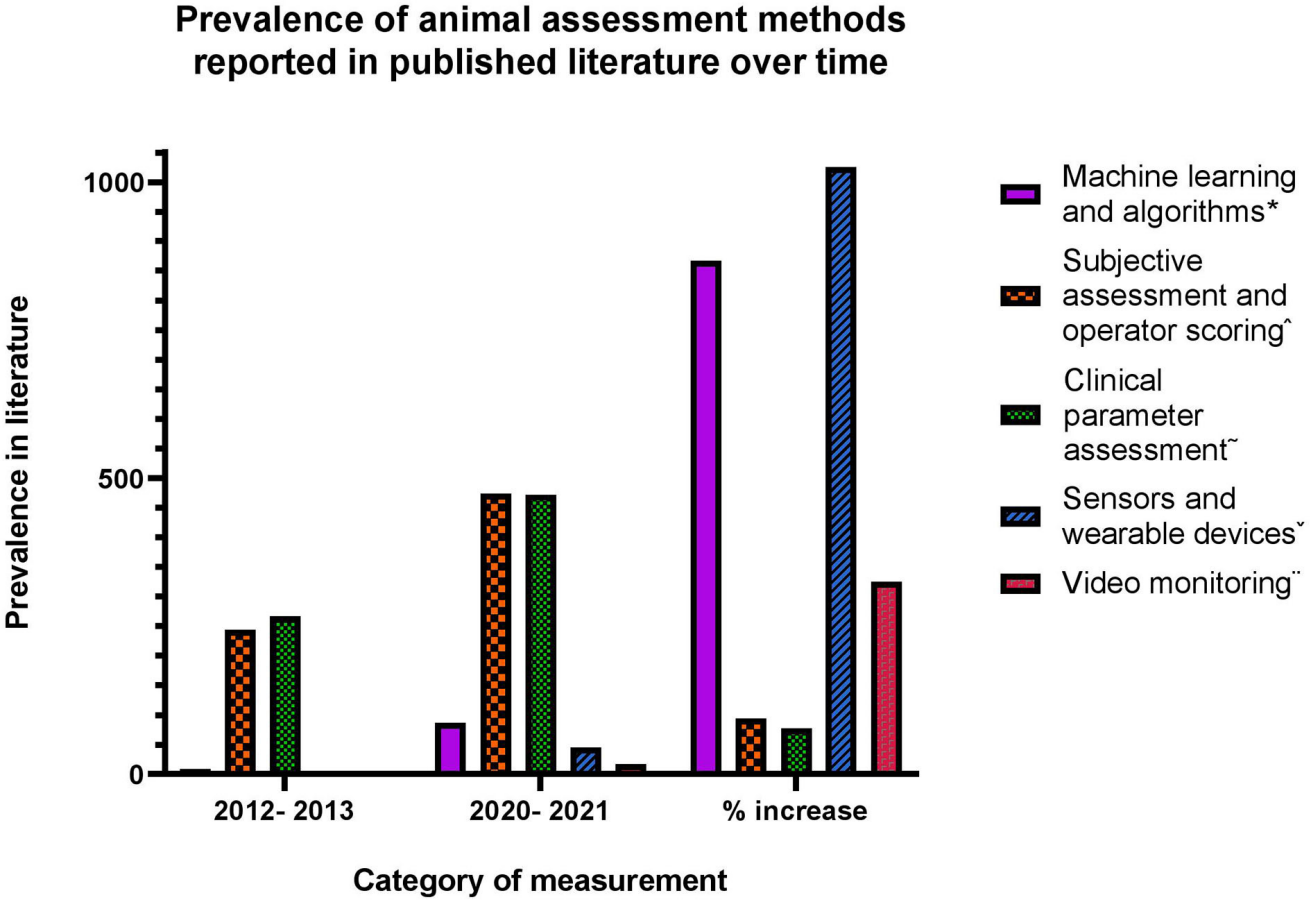
Data collected from Web of Science. Search refined by categories of Veterinary Sciences, Infectious Diseases, Agriculture multidisciplinary, Zoology. Search conducted of title, abstract, and key words using these terms per category: *Machine learning animal disease, algorithm animal disease. ^∧^Subjective assessment animal disease, clinical scoring animal disease, grimace score animal disease, clinical assessment animal disease. ^~^Heart rate animal disease, rectal temperature animal disease, blood pressure animal disease, respiration. ^∨^Sensors animal disease, wearable animal disease. ^¨^Video monitoring animal disease, infrared monitoring animal disease, motion detection monitoring animal disease.

To assess health, disease state and welfare in infectious disease animal research, a wide range of assessment methods are utilized (174). To better observe and understand the potential effects of stress and anesthesia, the methods of assessment and data collection used need to measure parameters with adequate sensitivity (175). In disease studies, a standardized approach to the assessment of health, disease and welfare state can be difficult to implement due to the large variation in the mechanisms of action and immune responses induced by the diseases being studied (176). Figure 4 describes methods of animal assessment commonly utilized in animal models of disease separated into five broad categories of assessment techniques, and demonstrates the change in focus of these methods of assessment over the past 7–9 years.

The increased prevalence in published literature of all methods of animal assessment in recent years is likely due to the heightened focus on animal welfare, leading to improved refinement of monitoring practices and increased reporting. Between 2012/2013 and 2020/2021, a search utilizing the same search parameters as used for Figure 4 shows a 48% increase in the term “welfare,” highlighting the increasing focus on animal welfare in research over the past 10 years. The specific broad categories of animal monitoring as represented in Figure 4 are explored in more detail below.

#### Subjective assessment and operator scoring

Subjective operator assessment of animals, for example using grimace scores and visual activity assessment, is widely utilized in animal studies of disease. The incidence of clinical assessment and operator scoring reported in the literature has increased by 96% between 2012/2013 to 2020/2021 (Figure 4). Of the five assessment categories discussed in this review, this category of assessment has seen the second lowest increase in recent years. Given the heightened focus on reporting of factors affecting research animal welfare, this increase is likely due to improved reporting in the literature, in addition to the increased use of this assessment method over time.

In recent years, the development of grimace scores has attempted to develop a more standardized approach to the assessment of pain in a range of laboratory species. These scoring systems were initially developed for laboratory mice but have since been expanded to a range of research animal species (177). Using the grimace score as a measure of pain and welfare in mice has resulted in an overall improvement and enhanced sensitivity for the assessment and detection of pain in a range of studies (178). More recently, Reijgwart et al. (179) compared facial musculature of ferrets pre- and post-surgery, to investigate and develop a ferret grimace score system. They found differences in facial musculature presentations and concluded that a ferret grimace score system could be useful in a multifactorial pain assessment (179). Similarly, a feline grimace score system has recently been developed by Evangelista et al. (180). They assessed cats post-operatively, and determined that the facial scoring system assessing ear position, orbital tightening, muzzle tension, whisker change, and head position was a valid and reliable tool for acute pain assessment in cats (180). Navarro et al. (181) developed a facial recognition scale for sows as a measure of pain, with observers reviewing photographs to score tension above eyes, snout angle, neck tension, temporal tension and ear angle. They determined that the scale was a useful tool for recognizing and assessing pain in farrowing sows, which indicates scope for employing such a facial pain assessment tool to further investigate its usefulness in infectious disease research in pigs (181). Benato et al. (182) expanded upon facial-based grimace scores by developing the Bristol rabbit pain score, encompassing demeanor, posture, locomotion, ears, eyes and grooming. A subsequent study by the authors where veterinary professionals used the scoring system to assess rabbits in acute pain determined it to be a suitable tool for quantifying pain in rabbits in a useful, valid and reliable way (183). One limitation of facial grimace scoring in infectious disease research is that clinical signs of disease that affect the face (eg, facial swelling in influenza) can make facial assessments less reliable (184). A pain recognition system such as the Bristol rabbit pain score may act as a more reliable pain measure in infectious disease research as it assesses more than just facial effects of pain, and should be investigated for use in infectious disease animal studies. There is scope to develop more holistic measures of pain and welfare assessment for laboratory animals in disease research that are more fit-for-purpose, with the potential to encompass facial, whole body and behavioral elements (185).

Behavioral scoring systems such as play and interaction scores are also commonly utilized in animal disease studies, which rely upon a visual assessment of activity level and behavior as judged by the assessor (186). These behavioral scoring systems are a useful tool in the identification and grading of disease impact, particularly for inquisitive and active species where changes in activity and behavior are readily apparent to the assessor. However, these scoring systems have limitations because they do not quantify the state of disease progression alone (187). They therefore must be utilized alongside the presence or absence of specific signs of disease for the particular disease model (188). This multi-faceted approach is an effective means of assessment of animals in disease studies, yet it is still prone to error and variability due to the inherent reliance on the subjective assessments of individuals. An understanding of the physiological mechanisms of the clinical signs exhibited can result in a more robust interpretation of health state, however the ultimate interpretation will depend on the assessor (188). The variability between research institutions will also vary as often the finer details of animal scoring systems and assessments are not published in the literature (189). This decreases the reproducibility of results in animal disease research. To enhance both scientific and welfare outcomes additional animal assessment methods should be implemented in infectious disease studies and details published, to complement existing subjective assessment methods.

#### Measurement of clinical parameters

Continual monitoring of clinical parameters such as body temperature and heart rate can be a useful means of data collection and health assessment in research animals (190). The prevalence of clinical parameter assessment in the literature has increased by 77% between 2012/2013 and 2020/2021 (Figure 4), which of the five categories of assessment discussed in this review is the lowest category to increase over the past 5 years. Due to enhanced reporting on welfare related aspects of animal studies, this increase could be reasonably attributed to an increase in the reporting of assessment methods, in addition to increased use to some degree. Whilst measurement of clinical parameters proves a useful monitoring strategy, it commonly requires the surgical implantation of telemetry devices where regular undisturbed data is required (191). The alternative is manual handling and disturbed measurements, which commonly leads to handling stress and artificially impacts clinical readouts (4).

Whilst the ability to collect this data *via* surgically implanted devices is invaluable in many disease studies, the effects of tissue trauma and surgery on the immune response has been well-categorized in the literature, with the strong consensus being that tissue damage from both trauma and surgery result in immunomodulation (38, 164, 192). Tissue damage caused by surgery results in the emission of large amounts of damage-associated molecular patterns, which induce a systemic cytokine and chemokine-mediated hyperinflammatory response (193). These responses typically result in immunoenhancement when these effects are acute, and immunosuppression when effects become chronic. However, hyperinflammation as a result of surgery can also result in acute tissue damage, resulting in increased disease susceptibility both acutely and chronically (170). This was demonstrated by Jia et al. (194) by conducting a meta-analysis of 25 articles, to investigate correlations between cytokine production capacity and the development of inflammatory complications post-surgery. The authors determined that elevated cytokine production capacity correlated with inflammatory complications post-surgery (194). This is consistent with previous theories that hyper-inflammation post-surgery triggers an anti-inflammatory compensatory immune response, causing immunosuppression and an increased risk of secondary complications (170). Whilst the degree of this response typically correlates with the degree of tissue damage, even minor surgery for telemetry implantation could be expected to result in a degree of hyperinflammation which, in addition to the welfare impacts of a surgical procedure, may affect disease study results (170).

The effects of a surgical procedure should therefore not be underestimated in animal disease studies and must be carefully weighed against the benefits of implanted telemetry devices. Using devices that capture multiple clinical parameters, as opposed to single measures like temperature alone, would assist in the risk/benefit assessment of surgically implanted devices, and result in better justification for the surgical procedures required for their use. Where surgical procedures are deemed important for achieving study outcomes, careful consideration of the time between surgery and disease induction should occur to minimize impacts on study objectives (195).

#### Sensors and wearable devices

Of the five categories of animal assessment and monitoring discussed in this review, the use of sensors and wearable devices has seen the most significant increase in published literature in recent years. From 2012/2013 to 2020/2021 the reported use of sensors and wearable devices has increased 1,025% (Figure 4), with the significance of the increase over time being as a result of limited reporting of this method in the literature using these search parameters in 2012–2013. This substantial increase strongly indicates a true increase in using these methods, more so than an increase in the reporting of assessment methods. Using sensors for physiological data collection in research has shown promise for the collection of some, but not all, metrics. González-Sánchez et al. (196) developed and trialed a circuit sensor system for the collection of heart activity and breathing pattern data using contactless sensors in mice, to avoid the need for restraint and sedation. Whilst they were able to collect breathing pattern data in a contactless manner, the system required a relatively complex set-up (196). This would likely prove to be an obstacle for many infectious disease studies, due to the restrictive nature of entry to rooms for troubleshooting complex technology (176). Equipment in disease studies must also be disposable or effectively decontaminated at the end of a study, and therefore complex equipment is often not well-suited or cost effective (176). In addition, González-Sánchez et al. (196) determined that heart activity could not be reliably monitored *via* the contactless system, and therefore required mice to stand on sensors to reliably collect data. Whilst still an effective means of data gathering that avoids the need for surgery, these results suggest that circuit sensor systems would not provide a practical and continual means of clinical parameter measurement in animal studies of infectious disease, and reliable data collection for multiple clinical parameters would still depend on anesthesia or handling.

In recent years, the uptake in wearable devices in the veterinary profession and amongst pet owners has increased (197). These devices typically involve sensor units attached to collars or bands for ease of use and a non-invasive means of monitoring clinical parameters. Heart rate variability (HRV) is being increasingly utilized for the measure of physiological and welfare state, and acts as a measure of cardiac autonomic modulation (198). The measurement of HRV can be conducted in various ways, including *via* the use of Holter-type monitors or electrocardiogram (199) using electrodes attached to the skin. Wearable monitors increasingly allow for these technologies to be used in a way that reduces the requirement for complex technological setups. These devices typically involve sensor units attached to collars or bands for ease of use. They can provide a non-invasive means of monitoring clinical parameters, including heart rate variability, and can be useful measures of pain (200) acute systemic inflammation (201) and stress (202). Despite the ease of use and apparent low welfare impacts, using wearable devices in animal disease research is not common practice. As with other technology-based data collection in disease research, complexity of set-up and use in the research setting, in addition to devices requiring disposal at the end of a study if they cannot be decontaminated, may be barriers to the uptake of wearable devices in animal disease research. In addition, the scarcity of published literature on the use of wearable devices in research animals may be a contributing factor to lack of validation of their effectiveness in off-label use for species commonly used in disease research.

A preliminary observational study by Paci et al. (203) demonstrated altered behaviors in a cat wearing a collar monitoring device compared to a control (no collar). Significant increases in grooming, scratching, biting, and head shaking were all observed with wearing of the collar, indicating discomfort. The authors propose that the focus on designing wearable data collection devices is on the user (human), more so than from the perspective of the animal wearing the device. For uptake of wearable devices in animal research, there is a need to ensure devices are indeed “non-invasive” from the experience of the animal. This ensures that wearability of devices is optimized, leading to real welfare benefits and optimized scientific outcomes by avoiding the introduction of altered behaviors that may influence study results. If these elements can be addressed, there is great promise in the ability to capture multi-parameter physiological data for the improvement of data collection, monitoring of animals, and refinement of humane endpoints in animal studies of infectious disease.

An area of research that has demonstrated successful development and uptake of wearable devices for physiological data collection is dairy cattle research. The successful implementation of wearable collars and devices in the research arena has led to uptake by dairy farmers for health management and production optimisation in dairy herds (204). For many other species used in research, the development of species-specific devices is likely not feasible due to a lack of market demand for such technology. The use and validation of devices already developed for production and companion animals may therefore be a cost-effective way to increase the uptake of wearable monitoring technologies in animal disease studies.

#### Video monitoring

Video monitoring has seen an increased prevalence in published literature of 325% between 2012/2013 and 2020/2021 (Figure 4). Whilst this is likely in part attributable to an increase in reporting of assessment methods, it also likely demonstrates a genuine increase in using video monitoring assessment. The desire to remove surgery (as required for implanted telemetry devices) as an experimental variable and improve upon welfare outcomes has more recently led to the advancement of less invasive vital sign and activity monitoring in research animals, *via* video-based assessments. As a result, the use and refinement of video monitoring in animal studies has increased significantly over the past decade, and is considered a useful tool for the non- or minimally invasive collection of behavioral data and clinical parameters in disease research (205).

Video monitoring systems are used in research to gather a wide range of metrics including respiratory rate, temperature *via* infra-red, heart rate, movement, and activity. These metrics can be a useful indicator of not only disease and inflammatory state, but also as a measure of pain and stress responses occurring *via* the autonomic nervous system (206). Infra-red thermography technology is being increasingly used in laboratory animal science to detect pain *via* skin surface vasoconstriction and vasodilation, in order to detect the effectiveness of analgesia and identify where pain may be causing confounding effects within studies (207). Optimized use of infra-red technology is dependent on a tailored approach to the species and study objectives. For example, pain and stress detection has been shown to be the most sensitive using ocular surfaces; aversive stimuli are more greatly detected *via* lowered tail and ear temperatures; and small mammal thermogenesis capacity can be most usefully measured *via* an interscapular window (208). The accuracy of infra-red detection can be affected by external factors such as wind speed, temperature, and humidity, requiring additional system processes to ensure consistency and accuracy of readings (209). Yet due to the commonly more controlled containment conditions of infectious disease research, the requirement for the control of these external variables is likely to be less. The presence, thickness and color of fur is another factor that can cause variability and lead to reduced reliability of readings (210). However, Loughin and Marino (211) determined that whilst the mean temperature of infra-red readings was lower in unshaved vs. shaved dogs, the thermal pattern was equally consistent. For infectious disease studies, where disease is commonly measured by comparing repeated measures throughout the disease course to healthy baseline data, the presence of fur would be unlikely to preclude the collection of valuable data (212). This was demonstrated by Schaefer et al. (213), who infected unshaved calves with type 2 bovine viral diarrhea virus and compared infra-red readings with unshaved, uninfected control calves. They found that infected calves displayed higher infra-red temperature readings prior to the onset of clinical disease or serum acute phase proteins, suggesting infra-red thermography successfully predicted clinical disease onset on calves (213).

Video systems vary significantly in cost and technological complexity, with simpler video setups commonly being restricted to temperature, movement, and activity monitoring. For the collection of these clinical parameters and metrics, video systems are an effective non-invasive method of collecting data on undisturbed animals. Yet the collection of additional parameters such as heart and respiratory rate generally requires a more technologically complex set-up and the requirement for animals to be still during measurement periods, leading to most studies using video monitoring for collection of heart and respiratory rate being conducted in anesthetized animals (214). In infectious disease studies, using video monitoring is uncommon, likely because of the need to anesthetize animals to gain a broad data set of clinical parameters. This presents additional safety risks to operators resulting from increased handling of infected animals, and an increased risk of sharps injury from parenteral anesthetic administration (215). Increasing the number of anesthetic inductions and/or time under anesthesia to measure clinical parameters *via* video systems also has potential detrimental effects on animal welfare and study outcomes, as already discussed in this review. Although anesthesia is not required or conducive for the collection of activity measurement, the measurement of activity in infectious disease studies is not common practice. This may be due to the technical aspects of activity monitoring *via* video capture, which commonly requires restrictive enclosure set-ups and for the body of the animal to represent much of the image (214). To overcome these issues, Oh et al. (216) temporarily placed individual ferrets infected with H1N1 and undergoing various antiviral treatments into a filming box daily. They found that video-tracking was more sensitive than manual behavior scoring in detecting activity changes, and that the video-tracking demonstrated that oseltamivir treatment alleviated the effect of influenza infection on activity in ferrets (216). These results demonstrate the value in incorporating the measurement of activity as a less subjective measure than operator assessments alone.

Overall, using video monitoring for the assessment of clinical parameters and activity metrics in animal research has progressed significantly in the past decade, yet the use of these technologies has not been adapted well to infectious disease research. Further studies into adapting technology to infectious disease research that is non-invasive and continuous, in addition to investigating the use of simpler technology that incorporates a range of measurements for real-time assessment, is required for the increased uptake of video monitoring in animal disease studies.

#### Machine learning and algorithms

There are a multitude of benefits to scientific outcomes and animal welfare by performing basic assessments of individual physical and physiological parameters in disease studies. However, the collection and assessment of individual metrics can be unreliable in capturing the complete disease and welfare state of an animal, and reliable predictions can be difficult to make (174). Therefore, a gold-standard approach to improving the assessment and prediction of disease outcomes is the incorporation of these metrics into machine learning algorithms (25). By feeding data of multiple physiological and behavioral metrics of a species into a machine learning program, algorithms can be developed that not only result in a more accurate assessment of overall health and disease state, but also allow for the prediction of short- and long-term outcomes of morbidity and mortality (217). By using algorithms in this way, animals can be identified as being at high risk of severe disease or death (218). Machine learning has also be utilized for pain and welfare assessment in animals, as has been done *via* the use of convolutional neural networks or facial action coding systems to detect pain in cats (219).

The development and use of machine learning algorithms in recent years, as described in Figure 4, has increased 867%. However, the small number of animal studies utilizing machine learning algorithms compared with other means of monitoring and assessment means that whilst a substantial percentage increase has occurred, this increase is inflated. The continued low relative use of this method of assessment demonstrates the significant potential for machine learning algorithms to be further adopted to improve animal welfare and scientific outcomes in disease research. An example of the benefits of machine learning algorithms is seen in a study by Mei et al. (220), who used published data from mouse models of stroke. They found large inter- and intra-model variance in humane endpoint determination and application due to varying animal models, lack of standardized experimental protocols, and heterogeneity of performance metrics (220). The authors then used previously published data on weight, temperature, and sickness scores from mouse models of sepsis and stroke and applied machine learning models to assess the usefulness of this method for parameter selection and endpoint definition across models. They found that the machine learning algorithms identified animals with a high risk of early death in both mouse models of stroke (male: 93.2% at 72 h post-treatment; female: 93.0% at 48 h post-treatment) and sepsis (96.2% at 24 h post-treatment), thus demonstrating generalizability of endpoint determination across models. Such research demonstrates the significant potential for machine learning algorithms in disease research, and strongly suggests the great potential of utilizing machine learning more broadly in animal models of infectious disease.

Hughes et al. (221) utilized machine learning and video footage to develop an algorithm for a model of Parkinson's disease in zebra fish. By improving the detection of early onset Parkinson's disease, they were able to improve data collection for the development of therapeutics (221). In addition, Ellmann et al. (222) developed a machine learning algorithm for the early detection of metastases in an experimental rat model, by utilizing various imaging data, tomography analysis and calculation of tumor-take rate. They found that the algorithm significantly outperformed the detection ability of each individual parameter, and that in addition the algorithm could be extrapolated for use with different organs or areas of multimodal and multiparametric imaging research (222). These studies further demonstrate both the feasibility and the potential for machine learning algorithms to be utilized more widely in animal disease research. In addition to this potential use of machine learning in future animal studies, there is also substantial opportunity for machine learning to be applied to existing data from previously conducted animal studies, which can then be refined as technology and the understanding of disease models advances.

Using behavioral and movement data collected *via* wearable sensors and video monitoring has also enabled the successful development of machine learning algorithms. Carslake et al. (223) equipped dairy calves with collar-mounted sensors and monitored their behavior with video cameras. They used sensor data and video observations to develop an algorithm to predict locomotor play behavior, which identified locomotor play (99.73% accuracy), self-grooming (98.18% accuracy), ruminating (94.47% accuracy), non-nutritive suckling (94.96% accuracy), nutritive suckling (96.44% accuracy), active lying (90.38% accuracy) and non-active lying (90.38% accuracy) (223). In animal disease research, the development of similar behavioral and movement algorithms would likely lead to an increased accuracy of detection of developing disease, by removing the subjectivity of operator assessments, combining several metrics, and allowing behavior and movement changes to be accurately detected over prolonged periods of time where animals are not disturbed by human presence.

The development and use of algorithms require specialist knowledge, in addition to the time investment of identifying and collecting the data required. However, the benefits of a significantly more accurate assessment of disease and improved predictions of humane endpoints holds great potential to improve both scientific outcomes and animal welfare. It is likely that the initial time investment to develop algorithms would lead to time savings in future studies utilizing the model by reducing the reliance on subjective operator assessments, and allowing for a more accurate and effective use of resources for animal monitoring. In addition, the potential for algorithms to be adapted between models would lead to further ease of development and application to a wider range of animal models in infectious disease research (224).

## Future directions

A holistic approach to study design and animal management practices in studies of infectious disease is crucial to reduce the physiological and immune impacts of stress, pain and anesthesia. Studies should firstly be designed to minimize the number of potentially stressful interventions required to achieve study objectives (such as sample collection events). To reduce the impacts of necessary interventions on animal welfare and scientific outcomes, further research is required to establish methods that reduce stress and reliance on anesthesia, whilst not introducing additional or unmanaged pain. This can undoubtedly be challenging in the field of infectious disease research due to the requirement for strict safety and biocontainment protocols. Yet small time investments in animal management techniques in studies during the animal habituation phase can reduce stress and the degree of chemical and physical restraint, as demonstrated in the use of training and restraint slings in research mini-pigs (225). The development of species-specific positive reinforcement training protocols, combined with multi-modal anesthesia regimes (such as local anesthetic with additional sedation to effect if required), tailored to the high biocontainment environment, should be explored as alternatives to forced manual handling and general anesthesia for repeated sample collections (226). Assessing and quantifying the impacts of new animal management regimes on animal welfare, scientific outcomes and staff safety, compared to methods that rely more heavily on manual and chemical restraint, is required to encourage the development and uptake of improved methods of conducting infectious disease research. The use and validation of a broader range of monitoring strategies and technologies, in particular minimally invasive wearable devices and video monitoring systems for the capture of physiological data, will aid in the comparative assessment of new methods (227). Additionally, data captured from the use of monitoring technologies will aid in an improved understanding of the diseases under investigation, and allow for a greater focus on machine learning and algorithm development (228). This has the potential to enhance our understanding of infectious disease processes, and improve animal welfare by refining animal monitoring strategies and refining humane endpoints in high-impact disease models.

## Conclusion

Stress and general anesthesia can result in reduced animal welfare and altered physiology and immunity. Both stress and general anesthesia have been found to cause immunomodulation, most commonly immunosuppression, which results in the introduction of variables in animal studies of infectious disease. Using general anesthesia in infectious disease research is integral where procedures or management practices cause undue or unmanaged pain or distress, but further research into improved management regimes for research animals is important to determine where anesthesia induction can be minimized without compromising animal welfare, increasing stress or causing pain and excessive inflammation to be poorly managed. Using monitoring technologies such as non-invasive wearable monitors, and the development of machine learning algorithms to better predict and manage disease and welfare, are required for a more multi-faceted monitoring approach in animal studies of infectious disease. This will lead to both a reduced reliance on subjective assessment measures and enhance our understanding of the effects of stress and anesthesia in disease studies. Further studies directly investigating the impacts of anesthesia and stress in infectious disease studies are required to improve research animal welfare and ensure greater science translatability from the laboratory to real world outcomes. This can be achieved by designing animal studies that use the least invasive techniques required to achieve study objectives; utilizing multi-modal anesthesia and analgesia to ensure pain, stress, and excessive inflammation are well-managed; and developing improved methods of animal management that result in less stress and a reduced reliance on anesthesia.

## Author contributions

RL wrote the manuscript with support from DL, DB, AF, PM, and KS. DB, AF, PM, and KS provide supervision. All authors contributed to the article and approved the submitted version.

## References

[1] Zusinaite E Ianevski A Niukkanen D Poranen MM Bjørås M Afset JE. A systems approach to study immuno- and neuro-modulatory properties of antiviral agents. Viruses. (2018) 10:423. 10.3390/v10080423 30103549 PMC6116047

[2] Swearengen JR. Choosing the right animal model for infectious disease research. Animal Model Exp Med. (2018) 1:100–8. 10.1002/ame2.12020 30891554 PMC6388060

[3] Gouveia K Hurst JL. Improving the practicality of using non-aversive handling methods to reduce background stress and anxiety in laboratory mice. Sci Rep. (2019) 9:20305. 10.1038/s41598-019-56860-7 31889107 PMC6937263

[4] Bailey J. Does the stress of laboratory life and experimentation on animals adversely affect research data? A critical review. Altern Lab Anim. (2018) 46:291–305. 10.1177/026119291804600501 30488713

[5] Bailoo JD Murphy E Boada-Saña M Varholick JA Hintze S Baussière C. Effects of cage enrichment on behavior, welfare and outcome variability in female mice. Front Behav Neurosci. (2018) 12:232. 10.3389/fnbeh.2018.00232 30416435 PMC6212514

[6] Petetta F Ciccocioppo R. Public perception of laboratory animal testing: historical, philosophical, and ethical view. Addict Biol. (2021) 26:e12991. 10.1111/adb.12991 33331099 PMC9252265

[7] Antoni MH Dhabhar FS. The impact of psychosocial stress and stress management on immune responses in patients with cancer. Cancer. (2019) 125:1417–31. 10.1002/cncr.31943 30768779 PMC6467795

[8] O'Connor DB Thayer JF Vedhara K. Stress and health: a review of psychobiological processes. Annu Rev Psychol. (2021) 72:663–88. 10.1146/annurev-psych-062520-122331 32886587

[9] Hennessy MB Willen RM Schiml PA. Psychological stress, its reduction, and long-term consequences: what studies with laboratory animals might teach us about life in the dog shelter. Animals. (2020) 10:2061. 10.3390/ani10112061 33171805 PMC7694980

[10] Williams DP Koenig J Carnevali L Sgoifo A Jarczok MN Sternberg EM. Heart rate variability and inflammation: a meta-analysis of human studies. Brain Behav Immun. (2019) 80:219–26. 10.1016/j.bbi.2019.03.009 30872091

[11] Wiley NC Dinan TG Ross RP Stanton C Clarke G Cryan JF. The microbiota-gut-brain axis as a key regulator of neural function and the stress response: implications for human and animal health. J Anim Sci. (2017) 95:3225–46. 10.2527/jas2016.1256 28727115

[12] Patchev VK Patchev AV. Experimental models of stress. Dialogues Clin Neurosci. (2006) 8:417–32. 10.31887/DCNS.2006.8.4/vpatchev 17290800 PMC3181831

[13] Van Dievel M Janssens L Stoks R. Short- and long-term behavioural, physiological and stoichiometric responses to predation risk indicate chronic stress and compensatory mechanisms. Oecologia. (2016) 181:347–57. 10.1007/s00442-015-3440-1 26385695

[14] Calefi AS Quinteiro-Filho WM Ferreira AJP Palermo-Neto J. Neuroimmunomodulation and heat stress in poultry. Worlds Poultry Sci J. (2017) 73:493–504. 10.1017/S0043933917000472

[15] Pietrelli A Di Nardo M Masucci A Brusco A Basso N Matkovic L. Lifelong aerobic exercise reduces the stress response in rats. Neuroscience. (2018) 376:94–107. 10.1016/j.neuroscience.2018.02.019 29462703

[16] Hernández-Avalos I Flores-Gasca E Mota-Rojas D Casas-Alvarado A Miranda-Cortés AE Domínguez-Oliva A. Neurobiology of anesthetic-surgical stress and induced behavioral changes in dogs and cats: a review. Vet World. (2021) 14:393–404. 10.14202/vetworld.2021.393-404 33776304 PMC7994130

[17] Dhabhar FS. Effects of stress on immune function: the good, the bad, and the beautiful. Immunol Res. (2014) 58:193–210. 10.1007/s12026-014-8517-0 24798553

[18] Lewis CE Pickering B. Livestock and risk group 4 pathogens: researching zoonotic threats to public health and agriculture in maximum containment. ILAR J. (2022) 61:86–102. 10.1093/ilar/ilab029 34864994 PMC8759435

[19] Ackerman RS Luddy KA Icard BE Piñeiro Fernández J Gatenby RA Muncey AR. The effects of anesthetics and perioperative medications on immune function: a narrative review. Anesth Analg. (2021) 133:676–89. 10.1213/ANE.0000000000005607 34100781

[20] Tian Y Guo S Wu X Ma L Zhao X. Minocycline alleviates sevoflurane-induced cognitive impairment in aged rats. Cell Mol Neurobiol. (2015) 35:585–94. 10.1007/s10571-014-0154-6 25585814 PMC11486178

[21] Tan XX Qiu LL Sun J. Research progress on the role of inflammatory mechanisms in the development of postoperative cognitive dysfunction. Biomed Res Int. (2021) 2021:3883204. 10.1155/2021/3883204 34869762 PMC8642009

[22] Gonçalves VC Pinheiro D de la Rosa T de Almeida AG Scorza FA Scorza CA. Propolis as a potential disease-modifying strategy in parkinson's disease: cardioprotective and neuroprotective effects in the 6-ohda rat model. Nutrients. (2020) 12:1551. 10.3390/nu12061551 32466610 PMC7352297

[23] Antanaitis R Juozaitiene V Malašauskiene D Televičius M Urbutis M Rutkaukas A. Identification of changes in rumination behavior registered with an online sensor system in cows with subclinical mastitis. Vet Sci. (2022) 9:454. 10.3390/vetsci9090454 36136670 PMC9503682

[24] Jorquera-Chavez M Fuentes S Dunshea FR Warner RD Poblete T Morrison RS. Remotely sensed imagery for early detection of respiratory disease in pigs: a pilot study. Animals. (2020) 10:451. 10.3390/ani10030451 32182745 PMC7142473

[25] Jirkof P Rudeck J Lewejohann L. Assessing affective state in laboratory rodents to promote animal welfare-what is the progress in applied refinement research? Animals. (2019) 9:1026. 10.3390/ani9121026 31775293 PMC6941082

[26] Baum A. Stress, intrusive imagery, and chronic distress. Health Psychol. (1990) 9:653–75. 10.1037/0278-6133.9.6.653 2286178

[27] Russell WMS Burch RL. The Principles of Humane Experimental Technique. London: Methuen (1959).

[28] Mota-Rojas D Miranda-Cortés A Casas-Alvarado A Mora-Medina P Boscato-Funes L Hernández-Ávalos I. Neurobiology and modulation of stress-induced hyperthermia and fever in animal. Abanico Vet. (2021) 11:21. 10.21929/abavet2021.1110721320

[29] Cohen S Janicki-Deverts D Doyle WJ Miller GE Frank E Rabin BS. Chronic stress, glucocorticoid receptor resistance, inflammation, and disease risk. Proc Natl Acad Sci U S A. (2012) 109:5995–9. 10.1073/pnas.1118355109 22474371 PMC3341031

[30] Zhou Q Qian Z Ding W Jiang G Sun C Xu K. Chronic psychological stress attenuates the efficacy of anti-Pd-L1 immunotherapy for bladder cancer in immunocompetent mice. Cancer Invest. (2021) 39:571–81. 10.1080/07357907.2021.1943746 34148483

[31] Gervasi SS Burgan SC Hofmeister E Unnasch TR Martin LB. Stress hormones predict a host superspreader phenotype in the west nile virus system. Proc Biol Sci. (2017) 284:20171090. 10.1098/rspb.2017.1090 28724737 PMC5543230

[32] Zhou Q Katano M Zhang JH Liu X Wang KY Iinuma M. Chewing behavior attenuates the tumor progression-enhancing effects of psychological stress in a breast cancer model mouse. Brain Sci. (2021) 11:479. 10.3390/brainsci11040479 33918787 PMC8069186

[33] Henderson LJ Dani B Serrano EMN Smulders TV Roughan JV. Benefits of tunnel handling persist after repeated restraint, injection and anaesthesia. Sci Rep. (2020) 10:14562. 10.1038/s41598-020-71476-y 32884048 PMC7471957

[34] Balcombe JP Barnard ND Sandusky C. Laboratory routines cause animal stress. Contemp Top Lab Anim Sci. (2004) 43:42–51. 15669134

[35] Jafari Z Kolb BE Mohajerani MH. Chronic traffic noise stress accelerates brain impairment and cognitive decline in mice. Exp Neurol. (2018) 308:1–12. 10.1016/j.expneurol.2018.06.011 29936225

[36] Marcon M Mocelin R Benvenutti R Costa T Herrmann AP de Oliveira DL. Environmental enrichment modulates the response to chronic stress in zebrafish. J Exp Biol. (2018) 221(Pt 4):jeb176735. 10.1242/jeb.176735 29361609

[37] Hernández-Avalos I Mota-Rojas D Mendoza-Flores JE Casas-Alvarado A Flores-Padilla K Miranda-Cortes AE. Nociceptive pain and anxiety in equines: physiological and behavioral alterations. Vet World. (2021) 14:2984–95. 10.14202/vetworld.2021.2984-2995 35017848 PMC8743789

[38] Desborough JP. The stress response to trauma and surgery. Br J Anaesth. (2000) 85:109–17. 10.1093/bja/85.1.109 10927999

[39] Kumar A Rinwa P Kaur G Machawal L. Stress: neurobiology, consequences and management. J Pharm Bioallied Sci. (2013) 5:91–7. 10.4103/0975-7406.111818 23833514 PMC3697199

[40] Jin Y Hu Y Han D Wang M. Chronic heat stress weakened the innate immunity and increased the virulence of highly pathogenic avian influenza virus H5n1 in mice. J Biomed Biotechnol. (2011) 2011:367846. 10.1155/2011/367846 21687549 PMC3114565

[41] Nicolaides NC Kyratzi E Lamprokostopoulou A Chrousos GP Charmandari E. Stress, the stress system and the role of glucocorticoids. Neuroimmunomodulation. (2015) 22:6–19. 10.1159/000362736 25227402

[42] Rohleder N. Stress and inflammation - the need to address the gap in the transition between acute and chronic stress effects. Psychoneuroendocrinology. (2019) 105:164–71. 10.1016/j.psyneuen.2019.02.021 30826163

[43] Clutton RE. An anglocentric history of anaesthetics and analgesics in the refinement of animal experiments. Animals. (2020) 10:1933. 10.3390/ani10101933 33096686 PMC7589666

[44] Kim R. Effects of surgery and anesthetic choice on immunosuppression and cancer recurrence. J Transl Med. (2018) 16:8. 10.1186/s12967-018-1389-7 29347949 PMC5774104

[45] Redondo JI Rubio M Soler G Serra I Soler C Gómez-Villamandos RJ. Normal values and incidence of cardiorespiratory complications in dogs during general anaesthesia. a review of 1281 cases. J Vet Med A Physiol Pathol Clin Med. (2007) 54:470–7. 10.1111/j.1439-0442.2007.00987.x 17931219

[46] Pottie RG Dart CM Perkins NR Hodgson DR. Effect of hypothermia on recovery from general anaesthesia in the dog. Aust Vet J. (2007) 85:158–62. 10.1111/j.1751-0813.2007.00128.x 17397389

[47] Vutskits L Xie Z. Lasting impact of general anaesthesia on the brain: mechanisms and relevance. Nat Rev Neurosci. (2016) 17:705–17. 10.1038/nrn.2016.128 27752068

[48] Bonhomme V Staquet C Montupil J Defresne A Kirsch M Martial C. General anesthesia: a probe to explore consciousness. Front Syst Neurosci. (2019) 13:36. 10.3389/fnsys.2019.00036 31474839 PMC6703193

[49] Numan T Slooter AJC van der Kooi AW Hoekman AML Suyker WJL Stam CJ. Functional connectivity and network analysis during hypoactive delirium and recovery from anesthesia. Clin Neurophysiol. (2017) 128:914–24. 10.1016/j.clinph.2017.02.022 28402867

[50] Kishikawa JI Inoue Y Fujikawa M Nishimura K Nakanishi A Tanabe T. General anesthetics cause mitochondrial dysfunction and reduction of intracellular ATP levels. PLoS ONE. (2018) 13:e0190213. 10.1371/journal.pone.0190213 29298324 PMC5752027

[51] Haas RH. Mitochondrial dysfunction in aging and diseases of aging. Biology. (2019) 8:48. 10.3390/biology8020048 31213034 PMC6627182

[52] Yang Y Liu Y Zhu J Song S Huang Y Zhang W. Neuroinflammation-mediated mitochondrial dysregulation involved in postoperative cognitive dysfunction. Free Radic Biol Med. (2022) 178:134–46. 10.1016/j.freeradbiomed.2021.12.004 34875338

[53] Tremoleda JL Kerton A Gsell W. Anaesthesia and physiological monitoring during *in vivo* imaging of laboratory rodents: considerations on experimental outcomes and animal welfare. EJNMMI Res. (2012) 2:44. 10.1186/2191-219X-2-44 22877315 PMC3467189

[54] Topal A Gül N Ilçöl Y Görgül OS. Hepatic effects of halothane, isoflurane or sevoflurane anaesthesia in dogs. J Vet Med A Physiol Pathol Clin Med. (2003) 50:530–3. 10.1111/j.1439-0442.2004.00589.x 15157022

[55] Lalonde S Truchetti G Otis C Beauchamp G Troncy E. Management of veterinary anaesthesia and analgesia in small animals: a survey of english-speaking practitioners in Canada. PLoS ONE. (2021) 16:e0257448. 10.1371/journal.pone.0257448 34582482 PMC8478190

[56] Cattai A Rabozzi R Ferasin H Isola M Franci P. Haemodynamic changes during propofol induction in dogs: new findings and approach of monitoring. BMC Vet Res. (2018) 14:282. 10.1186/s12917-018-1608-8 30208893 PMC6134702

[57] Mrazova M Rauser P Burova J Georgiou M Fichtel T. Influence of medetomidine, acepromazine, fentanyl and butorphanol on intraocular pressure and pupil size in healthy dogs. Vet Med. (2018) 63:413–9. 10.17221/51/2018-VETMED

[58] Brodbelt DC Blissitt KJ Hammond RA Neath PJ Young LE Pfeiffer DU. The risk of death: the confidential enquiry into perioperative small animal fatalities. Vet Anaesth Analg. (2008) 35:365–73. 10.1111/j.1467-2995.2008.00397.x 18466167

[59] Schuetze S Manig A Ribes S Nau R. Aged mice show an increased mortality after anesthesia with a standard dose of ketamine/xylazine. Lab Anim Res. (2019) 35:8. 10.1186/s42826-019-0008-y 32257896 PMC7081538

[60] Percie du Sert N Ahluwalia A Alam S Avey MT Baker M Browne WJ. Reporting animal research: explanation and elaboration for the arrive guidelines 2.0. PLoS Biol. (2020) 18:e3000411. 10.1371/journal.pbio.3000411 32663221 PMC7360025

[61] Begley CG Ioannidis JP. Reproducibility in science: improving the standard for basic and preclinical research. Circ Res. (2015) 116:116–26. 10.1161/CIRCRESAHA.114.303819 25552691

[62] Sessler DI. Perioperative thermoregulation and heat balance. Ann N Y Acad Sci. (1997) 813:757–77. 10.1111/j.1749-6632.1997.tb51779.x 9100967

[63] Rauch S Miller C Bräuer A Wallner B Bock M Paal P. Perioperative hypothermia-a narrative review. Int J Environ Res Public Health. (2021) 18:8749. 10.3390/ijerph18168749 34444504 PMC8394549

[64] Longley L. Anaesthesia and analgesia in rabbits and rodents. In Pract. (2008) 30:92–7. 10.1136/inpract.30.2.92

[65] Cicero L Fazzotta S Palumbo VD Cassata G Lo Monte AI. Anesthesia protocols in laboratory animals used for scientific purposes. Acta Biomed. (2018) 89:337–42. 10.23750/abm.v89i3.5824 30333456 PMC6502126

[66] Rodriguez-Diaz JM Hayes GM Boesch J Martin-Flores M Sumner JP Hayashi K. Decreased incidence of perioperative inadvertent hypothermia and faster anesthesia recovery with increased environmental temperature: a nonrandomized controlled study. Vet Surg. (2020) 49:256–64. 10.1111/vsu.13328 31617950

[67] Appenheimer MM Evans SS. Temperature and adaptive immunity. Handb Clin Neurol. (2018) 156:397–415. 10.1016/B978-0-444-63912-7.00024-2 30454603

[68] Centofanti P Barbero C D'Agata F Caglio MM Caroppo P Cicerale A. Neurologic and cognitive outcomes after aortic arch operation with hypothermic circulatory arrest. Surgery. (2016) 160:796–804. 10.1016/j.surg.2016.02.008 27048933

[69] Groene P Zeuzem C Baasner S Hofmann-Kiefer K. The influence of body mass index on temperature management during general anaesthesia-a prospective observational study. J Eval Clin Pract. (2019) 25:340–5. 10.1111/jep.13064 30450648

[70] Ruetzler K Kurz A. Consequences of perioperative hypothermia. Handb Clin Neurol. (2018) 157:687–97. 10.1016/B978-0-444-64074-1.00041-0 30459033

[71] Petrilli CM Jones SA Yang J Rajagopalan H O'Donnell L Chernyak Y. Factors associated with hospital admission and critical illness among 5279 people with coronavirus disease 2019 in New York City: Prospective Cohort Study. BMJ. (2020) 369:m1966. 10.1136/bmj.m1966 32444366 PMC7243801

[72] Khan F Tritschler T Kahn SR Rodger MA. Venous thromboembolism. Lancet. (2021) 398:64–77. 10.1016/S0140-6736(20)32658-1 33984268

[73] Wang X Du B Li J Wang S Wang X Guo M. D-Dimer surge and coagulation disorders in Covid-19 related pneumonia patients with cardiac injury: a case series. Medicine. (2020) 99:e21513. 10.1097/MD.0000000000021513 32756189 PMC7402888

[74] Iba T Levy JH Connors JM Warkentin TE Thachil J Levi M. The unique characteristics of Covid-19 coagulopathy. Critical Care. (2020) 24:360. 10.1186/s13054-020-03077-0 32552865 PMC7301352

[75] Vital signs in accidental hypothermia. High Alt Med Biol. (2021) 22:142–7. 10.1089/ham.2020.0179 33629884

[76] Li N Zhu L Sun L Shao G. The effects of novel coronavirus (SARS-CoV-2) infection on cardiovascular diseases and cardiopulmonary injuries. Stem Cell Res. (2021) 51:102168. 10.1016/j.scr.2021.102168 33485182 PMC7801189

[77] Nash PB. Susceptibility to low dose influenza in mice is increased by administration of ketamine/xylazine. J Immunol. (2021) 206(1 Supplement):177–196.

[78] Penna AM Johnson KJ Camilleri J Knight PR. Alterations in influenza a virus specific immune injury in mice anesthetized with halothane or ketamine. Intervirology. (1990) 31:188–96. 10.1159/000150153 2165044

[79] Cummings KA. Alpha-2 adrenergic agonists. In:Silverstein DC Rozanski EA Hopper K Drobatz KJ, editors. Textbook of Small Animal Emergency Medicine. Hoboken, NJ: John Wiley & Sons (2018), p. 1255–7. 10.1002/9781119028994.ch195

[80] Valverde A Skelding AM. Alternatives to opioid analgesia in small animal anesthesia: alpha-2 agonists. Vet Clin North Am Small Anim Pract. (2019) 49:1013–27. 10.1016/j.cvsm.2019.07.010 31481257

[81] Flanders CA Rocke AS Edwardson SA Baillie JK Walsh TS. The effect of dexmedetomidine and clonidine on the inflammatory response in critical illness: a systematic review of animal and human studies. Crit Care. (2019) 23:402. 10.1186/s13054-019-2690-4 31829277 PMC6907244

[82] Jain A Lamperti M Doyle DJ. Dexmedetomidine: another arrow in the quiver to fight COVID-19 in intensive care units. Br J Anaesth. (2021) 126:e35–e8. 10.1016/j.bja.2020.10.010 33190859 PMC7556802

[83] Yuki K. The immunomodulatory mechanism of dexmedetomidine. Int Immunopharmacol. (2021) 97:107709. 10.1016/j.intimp.2021.107709 33933842 PMC8324520

[84] Wang K Wu M Xu J Wu C Zhang B Wang G. Effects of dexmedetomidine on perioperative stress, inflammation, and immune function: systematic review and meta-analysis. Br J Anaesth. (2019) 123:777–94. 10.1016/j.bja.2019.07.027 31668347

[85] Cheng HW. The immunomodulatory effects of clonidine, an alpha-2-adrenergic agonist, in laying hens. Poult Sci. (2006) 85:452–6. 10.1093/ps/85.3.452 16553275

[86] Bournazos S Gupta A Ravetch JV. The role of Igg Fc receptors in antibody-dependent enhancement. Nat Rev Immunol. (2020) 20:633–43. 10.1038/s41577-020-00410-0 32782358 PMC7418887

[87] Wu Y Liu Y Huang H Zhu Y Zhang Y Lu F. Dexmedetomidine inhibits inflammatory reaction in lung tissues of septic rats by suppressing Tlr4/Nf-Kb pathway. Mediators Inflamm. (2013) 2013:562154. 10.1155/2013/562154 23690665 PMC3649753

[88] Zhang JJ Peng K Zhang J Meng XW Ji FH. Dexmedetomidine preconditioning may attenuate myocardial ischemia/reperfusion injury by down-regulating the Hmgb1-Tlr4-Myd88-Nf-Kb signaling pathway. PLoS ONE. (2017) 12:e0172006. 10.1371/journal.pone.0172006 28222157 PMC5319750

[89] Hamilton JL Vashi M Kishen EB Fogg LF Wimmer MA Balk RA. The association of an alpha-2 adrenergic receptor agonist and mortality in patients with Covid-19. Front Med. (2021) 8:797647. 10.3389/fmed.2021.797647 35059419 PMC8764306

[90] Moura E Afonso J Hein L Vieira-Coelho MA. Alpha2-adrenoceptor subtypes involved in the regulation of catecholamine release from the adrenal medulla of mice. Br J Pharmacol. (2006) 149:1049–58. 10.1038/sj.bjp.0706950 17075569 PMC2014633

[91] Giovannitti JA Jr Thoms SM Crawford JJ. Alpha-2 adrenergic receptor agonists: a review of current clinical applications. Anesth Prog. (2015) 62:31–9. 10.2344/0003-3006-62.1.31 25849473 PMC4389556

[92] Herrera-García AM Domínguez-Luis MJ Arce-Franco M Armas-González E Álvarez de La Rosa D Machado JD. Prevention of neutrophil extravasation by A2-adrenoceptor-mediated endothelial stabilization. J Immunol. (2014) 193:3023–35. 10.4049/jimmunol.1400255 25114107

[93] Lankadeva YR Shehabi Y Deane AM Plummer MP Bellomo R May CN. Emerging benefits and drawbacks of α -adrenoceptor agonists in the management of sepsis and critical illness. Br J Pharmacol. (2021) 178:1407–25. 10.1111/bph.15363 33450087

[94] Alonso B Carregaro A Cuypers C Michielsen A Gasthuys F Schauvliege S. Effects of detomidine or romifidine during maintenance and recovery from isoflurane anaesthesia in horses. Vet Anaesth Analg. (2022) 49:624–33. 10.1016/j.vaa.2022.07.004 36175293

[95] Flaherty D. Alpha2-adrenoceptor agonists in small animal practice 1. Why they do what they do. In Practice. (2013) 35:524–30. 10.1136/inp.f5826

[96] Connell AR Hookham MB Fu D Brazil DP Lyons TJ Yu JY. Comparisons of A2-adrenergic agents, medetomidine and xylazine, with pentobarbital for anesthesia: important pitfalls in diabetic and nondiabetic rats. J Ocul Pharmacol Ther. (2022) 38:156–66. 10.1089/jop.2021.0084 34964655 PMC8971989

[97] Zhu L She ZG Cheng X Qin JJ Zhang XJ Cai J. Association of blood glucose control and outcomes in patients with Covid-19 and pre-existing type 2 diabetes. Cell Metab. (2020) 31:1068–77.e3. 10.1016/j.cmet.2020.04.021 32369736 PMC7252168

[98] Dollery C. Therapeutic Drugs. London: Churchill Lingstone (1991), p. 1.

[99] Borba VV Zandman-Goddard G Shoenfeld Y. Prolactin and autoimmunity. Front Immunol. (2018) 9:73. 10.3389/fimmu.2018.00073 29483903 PMC5816039

[100] Liu GL Cui YF Lu C Zhao P. Ketamine a dissociative anesthetic: neurobiology and biomolecular exploration in depression. Chem Biol Interact. (2020) 319:109006. 10.1016/j.cbi.2020.109006 32084352

[101] Takahashi T Kinoshita M Shono S Habu Y Ogura T Seki S. The effect of ketamine anesthesia on the immune function of mice with postoperative septicemia. Anesth Analg. (2010) 111:1051–8. 10.1213/ANE.0b013e3181ed12fc 20705789

[102] Gao M Jin W Qian Y Ji L Feng G Sun J. Effect of N-methyl-D-aspartate receptor antagonist on T helper cell differentiation induced by phorbol-myristate-acetate and ionomycin. Cytokine. (2011) 56:458–65. 10.1016/j.cyto.2011.06.022 21795061

[103] Braun S Gaza N Werdehausen R Hermanns H Bauer I Durieux ME. Ketamine induces apoptosis *via* the mitochondrial pathway in human lymphocytes and neuronal cells. Br J Anaesth. (2010) 105:347–54. 10.1093/bja/aeq169 20659914

[104] Zeng J Xia S Zhong W Li J Lin L. *In vitro* and *in vivo* effects of ketamine on generation and function of dendritic cells. J Pharmacol Sci. (2011) 117:170–9. 10.1254/jphs.11113FP 22041942

[105] Laudanski K Qing M Oszkiel H Zawadka M Lapko N Nowak Z. Ketamine affects *in vitro* differentiation of monocyte into immature dendritic cells. Anesthesiology. (2015) 123:628–41. 10.1097/ALN.0000000000000783 26197043

[106] Eldufani J Nekoui A Blaise G. Nonanesthetic effects of ketamine: a review article. Am J Med. (2018) 131:1418–24. 10.1016/j.amjmed.2018.04.029 29753795

[107] Jafarzadeh A Hadavi M Hassanshahi G Rezaeian M Vazirinejad R. General anesthetics on immune system cytokines: a narrative review article. Anesth Pain Med. (2020) 10:e103033. 10.5812/aapm.103033 33134146 PMC7539048

[108] Li Y Shen R Wen G Ding R Du A Zhou J. Effects of ketamine on levels of inflammatory cytokines Il-6, Il-1β, and TNF-α in the hippocampus of mice following acute or chronic administration. Front Pharmacol. (2017) 8:139. 10.3389/fphar.2017.00139 28373844 PMC5357631

[109] Patten KT Valenzuela AE Wallis C Harvey DJ Bein KJ Wexler AS. Hippocampal but not serum cytokine levels are altered by traffic-related air pollution in TgF344-Ad and wildtype Fischer 344 rats in a sex- and age-dependent manner. Front Cell Neurosci. (2022) 16:861733. 10.3389/fncel.2022.861733 35530180 PMC9072828

[110] Banks WA. Peptides and the blood–brain barrier. Peptides. (2015) 72:16–9. 10.1016/j.peptides.2015.03.010 25805003 PMC5354301

[111] Liu FL Chen TL Chen RM. Mechanisms of ketamine-induced immunosuppression. Acta Anaesthesiol Taiwan. (2012) 50:172–7. 10.1016/j.aat.2012.12.001 23385040

[112] Steffey EP Mama KR Brosnan RJ. Inhalation anesthetics. In:Grimm K Lamont A Tranquilli J Greene S Robertson S, editors. Lumb & Jones' Veterinary Anesthesia. Hoboken, NJ: John Wiley & Sons, Inc (2015). p. 297–329. 10.1002/9781119421375.ch16

[113] Stollings LM Jia LJ Tang P Dou H Lu B Xu Y. Immune modulation by volatile anesthetics. Anesthesiology. (2016) 125:399–411. 10.1097/ALN.0000000000001195 27286478 PMC5074538

[114] Kalimeris K Christodoulaki K Karakitsos P Batistatou A Lekka M Bai M. Influence of propofol and volatile anaesthetics on the inflammatory response in the ventilated lung. Acta Anaesthesiol Scand. (2011) 55:740–8. 10.1111/j.1399-6576.2011.02461.x 21615348

[115] Woo JH Baik HJ Kim CH Chung RK Kim DY Lee GY. Effect of propofol and desflurane on immune cell populations in breast cancer patients: a randomized trial. J Korean Med Sci. (2015) 30:1503–8. 10.3346/jkms.2015.30.10.1503 26425050 PMC4575942

[116] Arruda NM Braz LG Nogueira FR Souza KM Aun AG Figueiredo DBS. Inflammation and DNA damage induction in surgical patients maintained with desflurane anesthesia. Mutat Res Genet Toxicol Environ Mutagen. (2019) 846:403073. 10.1016/j.mrgentox.2019.07.003 31585635

[117] Endo S Yano A Fukami T Nakajima M Yokoi T. Involvement of miRNAs in the early phase of halothane-induced liver injury. Toxicology. (2014) 319:75–84. 10.1016/j.tox.2014.02.011 24598351

[118] Khomich OA Kochetkov SN Bartosch B Ivanov AV. Redox biology of respiratory viral infections. Viruses. (2018) 10:392. 10.3390/v10080392 30049972 PMC6115776

[119] Kozlov EM Ivanova E Grechko AV Wu WK Starodubova AV Orekhov AN. Involvement of oxidative stress and the innate immune system in SARS-CoV-2 infection. Diseases. (2021) 9:17. 10.3390/diseases9010017 33668325 PMC8005963

[120] Erbas M Demiraran Y Yildirim HA Sezen G Iskender A Karagoz I. [Comparison of effects on the oxidant/antioxidant system of sevoflurane, desflurane and propofol infusion during general anesthesia]. Rev Bras Anestesiol. (2015) 65:68–72. 10.1016/j.bjane.2014.05.004 25443441

[121] Feng Y He X Yang Y Chao D Lazarus LH Xia Y. Current research on opioid receptor function. Curr Drug Targets. (2012) 13:230–46. 10.2174/138945012799201612 22204322 PMC3371376

[122] Pomorska DK Gach K Janecka A. Immunomodulatory effects of endogenous and synthetic peptides activating opioid receptors. Mini Rev Med Chem. (2014) 14:1148–55. 10.2174/1389557515666150101095237 25553430

[123] Chuang TK Killam KF Jr Chuang LF Kung HF Sheng WS Chao CC. Mu opioid receptor gene expression in immune cells. Biochem Biophys Res Commun. (1995) 216:922–30. 10.1006/bbrc.1995.2709 7488213

[124] Sacerdote P Franchi S Panerai AE. Non-analgesic effects of opioids: mechanisms and potential clinical relevance of opioid-induced immunodepression. Curr Pharm Des. (2012) 18:6034–42. 10.2174/138161212803582496 22747543

[125] Filipczak-Bryniarska I Nazimek K Nowak B Kozlowski M Wasik M Bryniarski K. In contrast to morphine, buprenorphine enhances macrophage-induced humoral immunity and, as oxycodone, slightly suppresses the effector phase of cell-mediated immune response in mice. Int Immunopharmacol. (2018) 54:344–53. 10.1016/j.intimp.2017.11.039 29197801

[126] Allen AA Kendall LV. Immunomodulation associated with sustained-release buprenorphine in female CD1 mice challenged with ovalbumin. J Am Assoc Lab Anim Sci. (2019) 58:577–82. 10.30802/AALAS-JAALAS-18-000135 31319903 PMC6774459

[127] Anderson SL Duke-Novakovski T Singh B. The immune response to anesthesia: part 2 sedatives, opioids, and injectable anesthetic agents. Vet Anaesth Analg. (2014) 41:553–66. 10.1111/vaa.12191 24962601

[128] Luan G Pan F Bu L Wu K Wang A Xu X. Butorphanol promotes macrophage phenotypic transition to inhibit inflammatory lung injury *via* K receptors. Front Immunol. (2021) 12:692286. 10.3389/fimmu.2021.692286 34305926 PMC8294090

[129] Shapouri-Moghaddam A Mohammadian S Vazini H Taghadosi M Esmaeili SA Mardani F. Macrophage plasticity, polarization, and function in health and disease. J Cell Physiol. (2018) 233:6425–40. 10.1002/jcp.26429 29319160 PMC13482560

[130] Widjaja G Turki Jalil A Sulaiman Rahman H Abdelbasset WK Bokov DO Suksatan W. Humoral immune mechanisms involved in protective and pathological immunity during Covid-19. Hum Immunol. (2021) 82:733–45. 10.1016/j.humimm.2021.06.011 34229864 PMC8245343

[131] Sacerdote P Bianchi M Manfredi B Panerai AE. Effects of tramadol on immune responses and nociceptive thresholds in mice. Pain. (1997) 72:325–30. 10.1016/S0304-3959(97)00055-9 9313273

[132] Saeed I La Caze A Hollmann MW Shaw PN Parat MO. New insights on tramadol and immunomodulation. Curr Oncol Rep. (2021) 23:123. 10.1007/s11912-021-01121-y 34448972

[133] Beilin B Grinevich G Yardeni IZ Bessler H. Tramadol does not impair the phagocytic capacity of human peripheral blood cells. Can J Anaesth. (2005) 52:1035–9. 10.1007/BF03021601 16326672

[134] Boland JW Foulds GA Ahmedzai SH Pockley AG. A preliminary evaluation of the effects of opioids on innate and adaptive human *in vitro* immune function. BMJ Support Palliat Care. (2014) 4:357–67. 10.1136/bmjspcare-2013-000573 24644198

[135] Wang ZY Wang CQ Yang JJ Sun J Huang YH Tang QF. Which has the least immunity depression during postoperative analgesia–morphine, tramadol, or tramadol with lornoxicam? Clin Chim Acta. (2006) 369:40–5. 10.1016/j.cca.2006.01.008 16487501

[136] Bianchi M Rossoni G Sacerdote P Panerai AE. Effects of tramadol on experimental inflammation. Fundam Clin Pharmacol. (1999) 13:220–5. 10.1111/j.1472-8206.1999.tb00342.x 10226767

[137] Bastami S Norling C Trinks C Holmlund B Walz TM Ahlner J. Inhibitory effect of opiates on LPS mediated release of TNF and IL-8. Acta Oncol. (2013) 52:1022–33. 10.3109/0284186X.2012.737932 23145506

[138] MacFater WS Xia W Barazanchi A Su'a B Svirskis D Hill AG. Intravenous local anaesthetic compared with intraperitoneal local anaesthetic in abdominal surgery: a systematic review. World J Surg. (2018) 42:3112–9. 10.1007/s00268-018-4623-9 29666908

[139] Taylor A McLeod G. Basic pharmacology of local anaesthetics. BJA Educ. (2020) 20:34–41. 10.1016/j.bjae.2019.10.002 33456928 PMC7808030

[140] Tavare AN Perry NJ Benzonana LL Takata M Ma D. Cancer recurrence after surgery: direct and indirect effects of anesthetic agents. Int J Cancer. (2012) 130:1237–50. 10.1002/ijc.26448 21935924

[141] Piegeler T Votta-Velis EG Liu G Place AT Schwartz DE Beck-Schimmer B. Antimetastatic potential of amide-linked local anesthetics: inhibition of lung adenocarcinoma cell migration and inflammatory SRC signaling independent of sodium channel blockade. Anesthesiology. (2012) 117:548–59. 10.1097/ALN.0b013e3182661977 22846676 PMC3482823

[142] Cassuto J Sinclair R Bonderovic M. Anti-inflammatory properties of local anesthetics and their present and potential clinical implications. Acta Anaesthesiol Scand. (2006) 50:265–82. 10.1111/j.1399-6576.2006.00936.x 16480459

[143] Kolle G Metterlein T Gruber M Seyfried T Petermichl W Pfaehler SM. Potential impact of local anesthetics inducing granulocyte arrest and altering immune functions on perioperative outcome. J Inflamm Res. (2021) 14:1–12. 10.2147/JIR.S275525 33442284 PMC7797324

[144] Cruz FF Rocco PR Pelosi P. Anti-inflammatory properties of anesthetic agents. Crit Care. (2017) 21:67. 10.1186/s13054-017-1645-x 28320449 PMC5359894

[145] Sheil M De Benedictis GM Scollo A Metcalfe S Innocent G Polkinghorne A. Efficacy of intra-operative topical wound anaesthesia to mitigate piglet castration pain-a large, multi-centred field trial. Animals. (2021) 11:2763. 10.3390/ani11102763 34679785 PMC8532673

[146] Milosavljevic SB Pavlovic AP Trpkovic SV Ilić AN Sekulic AD. Influence of spinal and general anesthesia on the metabolic, hormonal, and hemodynamic response in elective surgical patients. Med Sci Monit. (2014) 20:1833–40. 10.12659/MSM.890981 25284266 PMC4199462

[147] Edwards SH. Nonsteroidal Anti-Inflammatory Drugs in Animals. MSD Veterinary Manual, Vol. 11. Rahway, NJ: Merck & Co, Inc. (2022).

[148] Cashman JN. The mechanisms of action of nsaids in analgesia. Drugs. (1996) 52(Suppl 5):13–23. 10.2165/00003495-199600525-00004 8922554

[149] Schattenkirchner M. Meloxicam: a selective Cox-2 inhibitor non-steroidal anti-inflammatory drug. Expert Opin Investig Drugs. (1997) 6:321–34. 10.1517/13543784.6.3.321 15989631

[150] Michelin MA Figueiredo F Cunha FQ. Involvement of prostaglandins in the immunosuppression occurring during experimental infection by paracoccidioides brasiliensis. Exp Parasitol. (2002) 102:170–7. 10.1016/S0014-4894(03)00053-5 12856313

[151] Roth J Hübschle T Pehl U Ross G Gerstberger R. Influence of systemic treatment with cyclooxygenase inhibitors on lipopolysaccharide-induced fever and circulating levels of cytokines and cortisol in guinea-pigs. Pflugers Arch. (2002) 443:411–7. 10.1007/s004240100718 11810211

[152] Kalinski P. Regulation of immune responses by prostaglandin E2. J Immunol. (2012) 188:21–8. 10.4049/jimmunol.1101029 22187483 PMC3249979

[153] de Grauw JC van de Lest CH Brama PA Rambags BP van Weeren PR. *In vivo* effects of meloxicam on inflammatory mediators, mmp activity and cartilage biomarkers in equine joints with acute synovitis. Equine Vet J. (2009) 41:693–9. 10.2746/042516409X436286 19927589

[154] Kolstad AM Rodriguiz RM Kim CJ Hale LP. Effect of pain management on immunization efficacy in mice. J Am Assoc Lab Anim Sci. (2012) 51:448–57. 23043810 PMC3400693

[155] Fishback JE Stronsky SM Green CA Bean KD Froude JW. Antibody production in rabbits administered freund's complete adjuvant and carprofen concurrently. Lab Anim. (2016) 45:63–6. 10.1038/laban.937 26814352

[156] Kotiw M Morgan M Taylor SM Shiels IA. Detection of anti-tnfalpha activity in canine hyperimmune serum using a tnfalpha inhibition assay. Vet Clin Pathol. (2010) 39:46–52. 10.1111/j.1939-165X.2009.00166.x 19572976

[157] Hish GA Jr Diaz JA Hawley AE Myers DD Jr Lester PA. Effects of analgesic use on inflammation and hematology in a murine model of venous thrombosis. J Am Assoc Lab Anim Sci. (2014) 53:485–93. 25255071 PMC4181690

[158] DeMarco GJ Nunamaker EA. A review of the effects of pain and analgesia on immune system function and inflammation: relevance for preclinical studies. Comp Med. (2019) 69:520–34. 10.30802/AALAS-CM-19-000041 31896389 PMC6935697

[159] Giraudel JM Toutain PL King JN Lees P. Differential inhibition of cyclooxygenase isoenzymes in the cat by the nsaid robenacoxib. J Vet Pharmacol Ther. (2009) 32:31–40. 10.1111/j.1365-2885.2008.01031.x 19161453

[160] Bennett D Eckersall PD Waterston M Marchetti V Rota A McCulloch E. The effect of robenacoxib on the concentration of C-reactive protein in synovial fluid from dogs with osteoarthritis. BMC Vet Res. (2013) 9:42. 10.1186/1746-6148-9-42 23452411 PMC3610148

[161] Risser A Donovan D Heintzman J Page T. NSAID prescribing precautions. Am Fam Physician. (2009) 80:1371–8. 20000300

[162] Mbah C Ogbonna J Nzekwe I Ugwu G Ezeh R Builders P. Nanovesicle formulation enhances anti-inflammatory property and safe use of piroxicam. Pharm Nanotechnol. (2021) 9:177–90. 10.2174/2211738509666210129151844 33511937

[163] Pinho-Ribeiro FA Verri WA Jr Chiu IM. Nociceptor sensory neuron-immune interactions in pain and inflammation. Trends Immunol. (2017) 38:5–19. 10.1016/j.it.2016.10.001 27793571 PMC5205568

[164] Amodeo G Bugada D Franchi S Moschetti G Grimaldi S Panerai A. Immune function after major surgical interventions: the effect of postoperative pain treatment. J Pain Res. (2018) 11:1297–305. 10.2147/JPR.S158230 30022848 PMC6044362

[165] Page GG. The immune-suppressive effects of pain. Adv Exp Med Biol. (2003) 521:117–25. 12617570

[166] Peterson NC Nunamaker EA Turner PV. To treat or not to treat: the effects of pain on experimental parameters. Comp Med. (2017) 67:469–82. 29212578 PMC5713161

[167] Pezzone MA Dohanics J Rabin BS. Effects of footshock stress upon spleen and peripheral blood lymphocyte mitogenic responses in rats with lesions of the paraventricular nuclei. J Neuroimmunol. (1994) 53:39–46. 10.1016/0165-5728(94)90062-0 8051296

[168] Shavit Y Martin FC Yirmiya R Ben-Eliyahu S Terman GW Weiner H. Effects of a single administration of morphine or footshock stress on natural killer cell cytotoxicity. Brain Behav Immun. (1987) 1:318–28. 10.1016/0889-1591(87)90034-1 3453207

[169] Laudenslager ML Fleshner M Hofstadter P Held PE Simons L Maier SF. Suppression of specific antibody production by inescapable shock: stability under varying conditions. Brain Behav Immun. (1988) 2:92–101. 10.1016/0889-1591(88)90010-4 3148338

[170] Dabrowska AM Słotwiński R. Review paperthe immune response to surgery and infection. Cent Eur J Immunol. (2014) 39:532–7. 10.5114/ceji.2014.47741 26155175 PMC4439968

[171] Brown EN Pavone KJ Naranjo M. Multimodal general anesthesia: theory and practice. Anesth Analg. (2018) 127:1246–58. 10.1213/ANE.0000000000003668 30252709 PMC6203428

[172] Amaya V Paterson MBA Descovich K Phillips CJC. Effects of olfactory and auditory enrichment on heart rate variability in shelter dogs. Animals. (2020) 10:1385. 10.3390/ani10081385 32785115 PMC7460225

[173] Billman GE. The effect of heart rate on the heart rate variability response to autonomic interventions. Front Physiol. (2013) 4:222. 10.3389/fphys.2013.00222 23986716 PMC3752439

[174] Turner PV Pang DS Lofgren JL. A review of pain assessment methods in laboratory rodents. Comp Med. (2019) 69:451–67. 10.30802/AALAS-CM-19-000042 31896391 PMC6935698

[175] Klages C. IACUC and veterinary considerations for review of ABSL3 and ABSL4 research protocols. ILAR J. (2021) 61:3–9. 10.1093/ilar/ilab009 33782693 PMC8083656

[176] Kendall LV Owiny JR Dohm ED Knapek KJ Lee ES Kopanke JH. Replacement, refinement, and reduction in animal studies with biohazardous agents. ILAR J. (2018) 59:177–94. 10.1093/ilar/ily021 30668740

[177] Mota-Rojas D Olmos-Hernández A Verduzco-Mendoza A Hernández E Martínez-Burnes J Whittaker AL. The utility of grimace scales for practical pain assessment in laboratory animals. Animals. (2020) 10:1838. 10.3390/ani10101838 33050267 PMC7600890

[178] Langford DJ Bailey AL Chanda ML Clarke SE Drummond TE Echols S. Coding of facial expressions of pain in the laboratory mouse. Nat Methods. (2010) 7:447–9. 10.1038/nmeth.1455 20453868

[179] Reijgwart ML Schoemaker NJ Pascuzzo R Leach MC Stodel M de Nies L. The composition and initial evaluation of a grimace scale in ferrets after surgical implantation of a telemetry probe. PLoS ONE. (2017) 12:e0187986. 10.1371/journal.pone.0187986 29131858 PMC5683639

[180] Evangelista MC Watanabe R Leung VSY Monteiro BP O'Toole E Pang DSJ. Facial expressions of pain in cats: the development and validation of a feline grimace scale. Sci Rep. (2019) 9:19128. 10.1038/s41598-019-55693-8 31836868 PMC6911058

[181] Navarro E Mainau E Manteca X. Development of a facial expression scale using farrowing as a model of pain in sows. Animals. (2020) 10:2113. 10.3390/ani10112113 33202526 PMC7696890

[182] Benato L Murrell J Knowles TG Rooney NJ. Development of the Bristol Rabbit Pain Scale (BRPS): a multidimensional composite pain scale specific to rabbits (*Oryctolagus cuniculus*). PLoS ONE. (2021) 16:e0252417. 10.1371/journal.pone.0252417 34115781 PMC8195426

[183] Benato L Murrell J Rooney N. Bristol Rabbit Pain Scale (BRPS): clinical utility, validity and reliability. BMC Vet Res. (2022) 18:341. 10.1186/s12917-022-03434-x 36085033 PMC9461217

[184] Watanabe R Doodnaught GM Evangelista MC Monteiro BP Ruel HLM Steagall PV. Inter-rater reliability of the feline grimace scale in cats undergoing dental extractions. Front Vet Sci. (2020) 7:302. 10.3389/fvets.2020.00302 32548134 PMC7272704

[185] Hernandez-Avalos I Mota-Rojas D Mora-Medina P Martínez-Burnes J Casas Alvarado A Verduzco-Mendoza A. Review of different methods used for clinical recognition and assessment of pain in dogs and cats. Int J Vet Sci Med. (2019) 7:43–54. 10.1080/23144599.2019.1680044 31819890 PMC6882480

[186] Cleary SJ Pitchford SC Amison RT Carrington R Robaina Cabrera CL Magnen M. Animal models of mechanisms of SARS-CoV-2 infection and Covid-19 pathology. Br J Pharmacol. (2020) 177:4851–65. 10.1111/bph.15143 32462701 PMC7283621

[187] Denayer T Stöhr T Van Roy M. Animal models in translational medicine: validation and prediction. New Horiz Transl Med. (2014) 2:5–11. 10.1016/j.nhtm.2014.08.001

[188] Wemelsfelder F Mullan S. Applying ethological and health indicators to practical animal welfare assessment. Rev Sci Tech. (2014) 33:111–20. 10.20506/rst.33.1.2259 25000783

[189] Lilley E Stanford SC Kendall DE Alexander SPH Cirino G Docherty JR. Arrive 2.0 and the British Journal of Pharmacology: updated guidance for 2020. Br J Pharmacol. (2020) 177:3611–6. 10.1111/bph.15178 32662875 PMC7393193

[190] Mei J Riedel N Grittner U Endres M Banneke S Emmrich JV. Body temperature measurement in mice during acute illness: implantable temperature transponder versus surface infrared thermometry. Sci Rep. (2018) 8:3526. 10.1038/s41598-018-22020-6 29476115 PMC5824949

[191] Niemeyer JE. Telemetry for small animal physiology. Lab Anim. (2016) 45:255–7. 10.1038/laban.1048 27327012

[192] Carbone L. Pain in laboratory animals: the ethical and regulatory imperatives. PLoS ONE. (2011) 6:e21578. 10.1371/journal.pone.0021578 21915253 PMC3168441

[193] Relja B Land WG. Damage-associated molecular patterns in trauma. Eur J Trauma Emerg Surg. (2020) 46:751–75. 10.1007/s00068-019-01235-w 31612270 PMC7427761

[194] Jia R Zhou M Tuttle CSL Maier AB. Immune capacity determines outcome following surgery or trauma: a systematic review and meta-analysis. Eur J Trauma Emerg Surg. (2020) 46:979–91. 10.1007/s00068-019-01271-6 31781831 PMC7593308

[195] Huber-Lang M Lambris JD Ward PA. Innate immune responses to trauma. Nat Immunol. (2018) 19:327–41. 10.1038/s41590-018-0064-8 29507356 PMC6027646

[196] González-Sánchez C Fraile JC Pérez-Turiel J Damm E Schneider JG Zimmermann H. Capacitive sensing for non-invasive breathing and heart monitoring in non-restrained, non-sedated laboratory mice. Sensors. (2016) 16:1052. 10.3390/s16071052 27399713 PMC4970099

[197] Belda B Enomoto M Case BC Lascelles BDX. Initial evaluation of petpace activity monitor. Vet J. (2018) 237:63–8. 10.1016/j.tvjl.2018.05.011 30089547

[198] Halachmi I Guarino M Bewley J Pastell M. Smart animal agriculture: application of real-time sensors to improve animal well-being and production. Annu Rev Anim Biosci. (2019) 7:403–25. 10.1146/annurev-animal-020518-114851 30485756

[199] Hernández-Avalos I Valverde A Antonio Ibancovichi-Camarillo J Sánchez-Aparicio P Recillas-Morales S Rodríguez-Velázquez D. Clinical use of the parasympathetic tone activity index as a measurement of postoperative analgaesia in dogs undergoing ovariohysterectomy. J Vet Res. (2021) 65:117–23. 10.2478/jvetres-2021-0004 33817404 PMC8009586

[200] Rowlison de Ortiz A Belda B Hash J Enomoto M Robertson J Lascelles BDX. Initial exploration of the discriminatory ability of the petpace collar to detect differences in activity and physiological variables between healthy and osteoarthritic dogs. Front Pain Res. (2022) 3:949877. 10.3389/fpain.2022.949877 36147035 PMC9485802

[201] Magawa S Lear CA Beacom MJ King VJ Kasai M Galinsky R. Fetal heart rate variability is a biomarker of rapid but not progressive exacerbation of inflammation in preterm fetal sheep. Sci Rep. (2022) 12:1771. 10.1038/s41598-022-05799-3 35110628 PMC8810879

[202] Kézér FL Kovács L Tozsér J. Step behaviour and autonomic nervous system activity in multiparous dairy cows during milking in a herringbone milking system. Animal. (2015) 9:1393–6. 10.1017/S1751731115000130 25686697

[203] Paci P Mancini C Price BA. Designing for wearability in animal biotelemetry. In: Proceedings of the Third International Conference on Animal-Computer Interaction. Milton Keynes: Association for Computing Machinery (2016), p. 13. 10.1145/2995257.3012018

[204] Stygar AH Gómez Y Berteselli GV Dalla Costa E Canali E Niemi JK. A systematic review on commercially available and validated sensor technologies for welfare assessment of dairy cattle. Front Vet Sci. (2021) 8:634338. 10.3389/fvets.2021.634338 33869317 PMC8044875

[205] Richardson CA. The power of automated behavioural homecage technologies in characterizing disease progression in laboratory mice: a review. Appl Anim Behav Sci. (2015) 163:19–27. 10.1016/j.applanim.2014.11.018

[206] Casas-Alvarado A Mota-Rojas D Hernández-Ávalos I Mora-Medina P Olmos-Hernández A Verduzco-Mendoza A. Advances in infrared thermography: surgical aspects, vascular changes, and pain monitoring in veterinary medicine. J Therm Biol. (2020) 92:102664. 10.1016/j.jtherbio.2020.102664 32888567

[207] Mota-Rojas D Olmos-Hernández A Verduzco-Mendoza A Lecona-Butrón H Martínez-Burnes J Mora-Medina P. Infrared thermal imaging associated with pain in laboratory animals. Exp Anim. (2021) 70:1–12. 10.1538/expanim.20-0052 32848100 PMC7887630

[208] Verduzco-Mendoza A Bueno-Nava A Wang D Martínez-Burnes J Olmos-Hernández A Casas A. Experimental applications and factors involved in validating thermal windows using infrared thermography to assess the health and thermostability of laboratory animals. Animals. (2021) 11:3448. 10.3390/ani11123448 34944225 PMC8698170

[209] Wang FK Shih JY Juan PH Su YC Wang YC. Non-invasive cattle body temperature measurement using infrared thermography and auxiliary sensors. Sensors. (2021) 21:2425. 10.3390/s21072425 33915906 PMC8037298

[210] Rekant SI Lyons MA Pacheco JM Arzt J Rodriguez LL. Veterinary applications of infrared thermography. Am J Vet Res. (2016) 77:98–107. 10.2460/ajvr.77.1.98 26709943

[211] Loughin C Marino D. Evaluation of thermographic imaging of the limbs of healthy dogs. Am J Vet Res. (2007) 68:1064–9. 10.2460/ajvr.68.10.1064 17916011

[212] Warren-Gash C Blackburn R Whitaker H McMenamin J Hayward AC. Laboratory-confirmed respiratory infections as triggers for acute myocardial infarction and stroke: a self-controlled case series analysis of national linked datasets from Scotland. Eur Respir J. (2018) 51:170179. 10.1183/13993003.01794-2017 29563170 PMC5898931

[213] Schaefer AL Cook N Tessaro SV Deregt D Desroches G Dubeski PL. Early detection and prediction of infection using infrared thermography. Can J Anim Sci. (2004) 84:73–80. 10.4141/A02-104

[214] Pereira C Kunczik J Bleich A Haeger C Kiessling F Thum T. Perspective review of optical imaging in welfare assessment in animal-based research. J Biomed Opt. (2019) 24:1–11. 10.1117/1.JBO.24.7.070601 31286726 PMC6995877

[215] Homer LC Alderman TS Blair HA Brocard AS Broussard EE Ellis RP. Guidelines for Biosafety training programs for workers assigned to BSL-3 research laboratories. Biosecur Bioterror. (2013) 11:10–9. 10.1089/bsp.2012.0038 23477631

[216] Oh DY Barr IG Hurt AC. A novel video tracking method to evaluate the effect of influenza infection and antiviral treatment on ferret activity. PLoS ONE. (2015) 10:e0118780. 10.1371/journal.pone.0118780 25738900 PMC4349809

[217] Wu B Abbott T Fishman D McMurray W Mor G Stone K. Comparison of statistical methods for classification of ovarian cancer using mass spectrometry data. Bioinformatics. (2003) 19:1636–43. 10.1093/bioinformatics/btg210 12967959

[218] Price A Okumura A Haddock E Feldmann F Meade-White K Sharma P. Transcriptional correlates of tolerance and lethality in mice predict ebola virus disease patient outcomes. Cell Rep. (2020) 30:1702–13.e6. 10.1016/j.celrep.2020.01.026 32049004 PMC11062563

[219] Feighelstein M Shimshoni I Finka LR Luna SPL Mills DS Zamansky A. Automated recognition of pain in cats. Sci Rep. (2022) 12:9575. 10.1038/s41598-022-13348-1 35688852 PMC9187730

[220] Mei J Banneke S Lips J Kuffner MTC Hoffmann CJ Dirnagl U. Refining humane endpoints in mouse models of disease by systematic review and machine learning-based endpoint definition. ALTEX. (2019) 36:555–71. 10.14573/altex.1812231 31026040

[221] Hughes GL Lones MA Bedder M Currie PD Smith SL Pownall ME. Machine learning discriminates a movement disorder in a zebrafish model of Parkinson's disease. Dis Model Mech. (2020) 13:dmm045815. 10.1242/dmm.045815 32859696 PMC7578351

[222] Ellmann S Seyler L Gillmann C Popp V Treutlein C Bozec A. Machine learning algorithms for early detection of bone metastases in an experimental rat model. J Vis Exp. (2020) 162. 10.3791/61235 32865533

[223] Carslake C Vázquez-Diosdado JA Kaler J. Machine learning algorithms to classify and quantify multiple behaviours in dairy calves using a sensor: moving beyond classification in precision livestock. Sensors. (2020) 21:88. 10.3390/s21010088 33375636 PMC7795166

[224] Gu Y Ge Z Bonnington CP Zhou J. Progressive transfer learning and adversarial domain adaptation for cross-domain skin disease classification. IEEE J Biomed Health Inform. (2020) 24:1379–93. 10.1109/JBHI.2019.2942429 31545748

[225] O'Malley CI Hubley R Tambadou H Turner PV. Refining restraint techniques for research pigs through habituation. Front Vet Sci. (2022) 9:1016414. 10.3389/fvets.2022.1016414 36213394 PMC9541109

[226] Chiesa OA Gonzales R Kouneski A Lewandowski A Rotstein D Myers MJ. Minimally invasive ultrasound-guided technique for central venous catheterization *via* the external jugular vein in pigs. Am J Vet Res. (2021) 82:760–9. 10.2460/ajvr.82.9.760 34432513

[227] Ortmeyer HK Robey L McDonald T. Combining actigraph link and PetPace collar data to measure activity, proximity, and physiological responses in freely moving dogs in a natural environment. Animals. (2018) 8:230. 10.3390/ani8120230 30518086 PMC6316215

[228] Taneja M Byabazaire J Jalodia N Davy A Olariu C Malone P. Machine learning based fog computing assisted data-driven approach for early lameness detection in dairy cattle. Comput Electron Agric. (2020) 171:105286. 10.1016/j.compag.2020.105286

